# The proteomic landscape of diffuse midline glioma highlights the therapeutic potential of non-histone protein methyltransferases

**DOI:** 10.1093/neuonc/noaf033

**Published:** 2025-02-15

**Authors:** Arun Kumaran Anguraj Vadivel, Sanja Pajovic, Robert Siddaway, Sabrina Zhu, Stefanie-Grace Sbergio, Olivera Matic, Lauren Phillips, Yong Jia Bu, Mark Nitz, Cynthia Hawkins

**Affiliations:** The Arthur and Sonia Labatt Brain Tumour Research Centre, The Hospital for Sick Children, Toronto, Ontario, Canada; The Arthur and Sonia Labatt Brain Tumour Research Centre, The Hospital for Sick Children, Toronto, Ontario, Canada; Division of Pathology, The Hospital for Sick Children, Toronto, Ontario, Canada; Department of Laboratory Medicine and Pathobiology, University of Toronto, Toronto, Ontario, Canada; Department of Laboratory Medicine and Pathobiology, University of Toronto, Toronto, Ontario, Canada; The Arthur and Sonia Labatt Brain Tumour Research Centre, The Hospital for Sick Children, Toronto, Ontario, Canada; The Arthur and Sonia Labatt Brain Tumour Research Centre, The Hospital for Sick Children, Toronto, Ontario, Canada; The Arthur and Sonia Labatt Brain Tumour Research Centre, The Hospital for Sick Children, Toronto, Ontario, Canada; Department of Chemistry, University of Toronto, Toronto, Ontario, Canada; Department of Chemistry, University of Toronto, Toronto, Ontario, Canada; Division of Pathology, The Hospital for Sick Children, Toronto, Ontario, Canada; Department of Laboratory Medicine and Pathobiology, University of Toronto, Toronto, Ontario, Canada; The Arthur and Sonia Labatt Brain Tumour Research Centre, The Hospital for Sick Children, Toronto, Ontario, Canada

**Keywords:** DMG, DIPG, METTL13, non-histone methyltransferase, proteomics

## Abstract

**Background:**

Diffuse midline glioma (DMG) is a highly aggressive pediatric brain tumor with limited treatment options despite extensive genomic characterization. The aim of this study was to investigate the proteomic landscape of DMG to identify potential therapeutic targets.

**Methods:**

We conducted a comprehensive proteomic analysis using LC-MS^3^, along with DNA methylation and DNA/RNA sequencing in 55 DMG patients’ samples. post-translational modification profiling (phosphoproteome and methylproteome) was conducted in 30 patient samples. We then investigated the effects of modulating key protein targets on protein methylation, protein synthesis, and DMG cell growth *in vitro* and *in vivo*.

**Results:**

DMGs exhibited high global protein methylation, with significant enrichment of translation machinery proteins and factors involved in apoptosis regulation. Surprisingly, while targets of key kinases were highly phosphorylated, overall protein phosphorylation was lower in DMG compared to normal brain tissues. Non-histone methyltransferases METTL13 and METTL21B, along with protein kinases PAK2, PRKACA, and AKT1, were identified as key players in DMG methylproteome and phosphoproteome, respectively. METTL13 knockdown led to reduced EEF1A1 protein methylation, a shift in oncoprotein synthesis, and inhibited DMG cell growth *in vitro* and *in vivo*.

**Conclusions:**

Our findings highlight the dependency of DMG on methyl-signaling pathways, particularly involving METTL13, which regulates EEF1A1 protein methylation and oncoprotein synthesis. Targeting the non-histone methyltransferases offers a promising therapeutic strategy for DMG. This study underscores the potential of post-translational modifications, specifically methyl-signaling pathways, as novel therapeutic targets for DMG and possibly other currently incurable cancers.

## Graphical Abstract

**Graphical Abstract:**
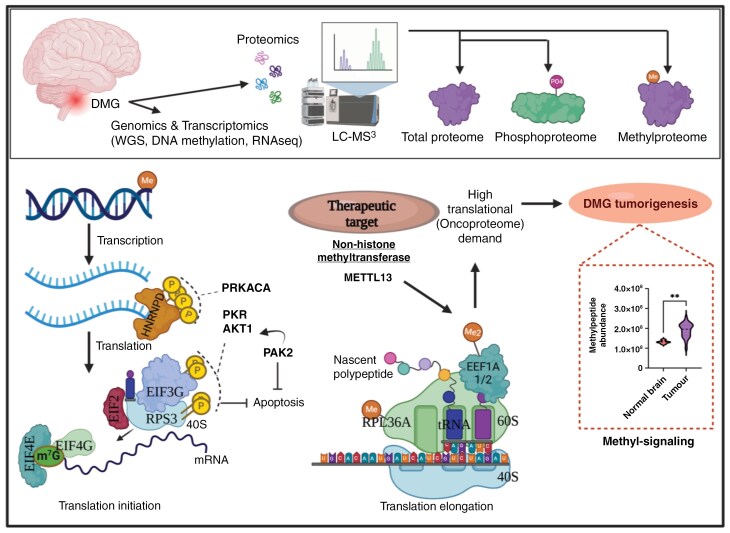

**Key Points**

- DMGs depend on methyl-signaling for tumor growth, showing high global protein methylation.
- METTL13, METTL21B, PAK2, PRKACA, and AKT1 are important players in DMG pathogenesis.
- EEF1A1 protein methylation is associated with poor prognosis in DMG patients.

**Importance of the Study**

This study provides critical insights into the molecular underpinnings of diffuse midline glioma (DMG), an aggressive and difficult-to-treat pediatric brain tumor. By conducting a comprehensive proteomic analysis, we identified a significant dependency of DMGs on methyl-signaling pathways, particularly highlighting the role of non-histone methyltransferase METTL13. The discovery that this methyltransferase regulates EEF1A1 protein methylation and specifically oncoprotein synthesis underscores its potential as a novel therapeutic target. Additionally, the identification of key protein kinases (PAK2, PRKACA, and AKT1) involved in DMG pathogenesis offers further avenues for targeted treatments. This study advances our understanding of DMG biology and opens new possibilities for therapeutic interventions, which is crucial given the limited effective treatment options currently available. Targeting post-translational modifications, specifically methyl-signaling pathways, could lead to innovative treatments for DMG and potentially other incurable cancers, significantly impacting patient outcomes.

Sequencing studies over the last decade identified highly recurrent lysine 27 to methionine (K27M) substitutions in histones H3.3 or H3.1, largely restricted to midline glial neoplasms.^1–4^ These are particularly common in children where they most typically arise in the pons and have traditionally been called diffuse intrinsic pontine glioma. With the finding of H3K27M mutations, these tumors have been increasingly recognized in adults and in other midline regions such as the thalamus or spinal cord. Given this, the most recent World Health Organization classification of CNS tumors has consolidated this group as DMG, H3K27-altered (diffuse midline glioma [DMG]). DMG is an incurable, extremely aggressive, diffusely infiltrative brain tumor.^5,6^ The unmet clinical need for effective DMG treatments is urgent and the limitations of current therapies, such as radiation and chemotherapy, have emphasized the necessity to explore novel therapeutic strategies for this devastating disease. While DMG has undergone thorough genetic profiling, its study at the proteome and post-translational modification (PTM) levels, which are more proximal to potential drug therapy targets, remains limited.^7–9^ Combining genomic, transcriptomic, and proteomic data has been particularly powerful in uncovering relevant cancer subtypes and new druggable targets.^10–13^ Furthermore, despite extensive exploration of protein phosphorylation signaling in cancer,^14–17^ the vast landscape of global protein methylation, covering both lysine and arginine residues, remains uncharted in cancer patients.^18^ The ability to systematically track lysine and arginine methylation throughout the proteome, with a specific emphasis on non-histone proteins, in a quantitative fashion using newer mass spectrometry technologies enables a new understanding of the role of protein methylation in oncogenic signaling.

In this study, we utilized advanced mass spectrometry to profile the total proteome, phosphoproteome, and methylproteome of DMG generating a new understanding of DMG pathogenesis which was lacking from RNA and DNA level studies. We identified PRKACA, PAK2, and AKT1 as key mediators of the DMG phosphoproteome while METTL13 and METTL21B are key drivers of the DMG methylproteome. We show that global protein methylation is significantly elevated in DMG compared to normal brains, suggesting that methyl-signaling is a hitherto unappreciated pro-oncogenic driver of DMG. Importantly targeting DMG-enriched methyltransferase METTL13 altered the oncoproteome and reduced DMG cell growth *in vitro* and in patient-derived xenograft models *in vivo*. These data highlight the importance of the methylproteome in oncogenesis and its promise for identifying novel therapeutic targets in incurable cancers like DMG.

## Methods

### Ethical Approval and Patient Samples

All autopsies were performed with consent from legal guardians/next of kin using the hospital approved autopsy consent which included a consent to use the collected samples for research purposes. Use of these samples for research purposes was approved by The Hospital for Sick Children’s Research Ethics Board (#1000055059).

### DMG Samples

#### Multi-omics cohort.—

39 DMG samples (27 H3.3K27M, 6 H3.1K27M, 6 H3WT) and 16 normal brain autopsy tissues were used for DNA methylation profiling, bulk RNA sequencing, and total proteome analysis. Detailed information on the cohort is available in Supplementary Table 1.

#### PTM Cohort.—

24 DMG samples and 6 normal brain autopsy tissues were used for phosphoproteome and methylproteome analysis. Due to limited sample availability, only these cohort samples were used for PTM profiling and integrative analysis with total proteome, RNAseq, and DNA methylation data.

All samples used in this study were derived from autopsy tissues and have high tumor content based on examination of matched H&E staining and H3K27M mutant variant allele frequency (VAF 0.3-0.6 indicating 60 to close to 100% tumor content). Postmortem intervals ranged from 8 to 60 hours and there was no correlation between total phosphopeptides and postmortem interval in this time range (R2 = 0.001).

### WGS/WES

Whole genome sequencing (WGS) and whole exome sequencing (WES) were taken from previously published data on our patient cohort,^19–21^ as described in the data availability section.

### Protein Extraction, Trypsin/LysC Digestion, and TMT-Labeling

For proteomic analysis of samples, 2% sodium dodecyl sulfate (SDS) lysis buffer (100 mM (4-(2-hydroxyethyl)-1-piperazineethanesulfonic acid; HEPES) pH 7.3, 2% SDS, 50 mM NaCl, 10 mM Tris(2-carboxyethyl)phosphine (TCEP), 40 mM Chloroacetamide (CAA), and complete protease inhibitor) was used to quickly lyse cells from cell culture or from tumor samples by heating at 95 °C for 15 minutes. 50 μg of proteins were precipitated by the methanol-chloroform method and digested with 1 μg trypsin/Lys-C mixture (Promega).

In total, 25 μg of peptides of indicated samples were labeled with 10plex Mass Tag Labeling Kits according to the manufactory manual (ThermoScientific). Ten samples as indicated in each experiment were pooled after labeling and fractionated by Pierce high pH kit (Pierce™). Sixty fractions of each TMT labeled sample were then analyzed by Thermo Orbitrap Fusion Lumos Tribrid Mass Spectrometer.

### Phosphopeptide Enrichment

Phosphopeptides were enriched using Pierce™ Phosphoprotein Enrichment Kit (Thermo Scientific) by following the user manual. The enriched peptides were analyzed along with total protein samples by Thermo Orbitrap Fusion Lumos Tribrid Mass Spectrometer.

### SCX-LC-MS^3^ (Methylproteome Profiling)

Protein extracts from DMG cells were trypsin digested and processed by high pH strong cation exchange (SCX) to profile methylproteome of the cells. In brief, 0.5 mg of digested protein was resuspended in loading buffer (60% acetonitrile, 40% BRUB; 5 mM phosphoric acid, 5 mM boric acid, 5 mM acetic acid, pH 2.5) and incubated with high pH SCX beads (Sepax, Newark, DE) for 30 minutes, washed with washing buffer (80% acetonitrile, 20% BRUB, pH 9), and eluted into 5 fractions using elution buffer 1 (60% acetonitrile, 40% BRUB, pH 9), elution buffer 2 (60% acetonitrile, 40% BRUB, pH 10), elution buffer 3 (60% acetonitrile, 40% BRUB, pH 11), elution buffer 4 (30% acetonitrile, 70% BRUB, pH 12), and elution buffer 5 (100% BRUB, 1 M NaCl, pH12). Eluates were dried and resuspended in 1% trifluoroacetic acid.

### LC-MS^3^

For MS analysis of proteins, peptides were separated by reverse-phase chromatography using a nanoflow Ultra-high-performance liquid chromatography (UPLC) system (ThermoFisher Scientific) with a linear gradient. UPLC was coupled online to an Orbitrap Elite, Q-Exactive, or an Orbitrap Lumos MS platform (ThermoScientific). Data acquisition was conducted using a data-dependent method with multinotch synchronous precursor selection and MS^3^ scanning for TMT tags.

Raw files were analyzed on Proteome Discover utilizing both SequestHT and MSAmanda 2.0 search algorithms and searched against Uniprot database (Uniprot_UP000005640_Human_15092020.fasta downloaded September 15, 2020, 74 854 entries). Proteins with a 1% false discovery rate (FDR) confidence were considered for further analysis. For total, phospho, and methylpeptides, we had 3 sets of TMT-labeled samples, ran as 60 fraction per TMT set, 1 hour per fraction. The peptides from each fraction were pooled and analyzed.. Fractionation of the pooled samples (1TMT set = 25 ug × 10 samples = 250 ug/60 fractions = ~ 4 ug/fraction, loaded ½ so ~2 ug per fraction) allows for the highly complex mixtures to be spread out over 60 MS runs. On average, ~30 000 peptides per TMT set were identified with a total of 88 965 peptides for total TMT sets and then assigned to each sample based on TMT-tags by default by Proteome Discoverer. Protein abundance or protein intensity is defined as the sum of all peptide intensities corresponding to a given protein. Quantification was performed using TMT-MS3 reporter ion intensities. Specifically, the ratio of reporter ion intensities across tissues was calculated for each peptide, and these ratios were combined to derive protein-level quantification. The resulting protein intensities were then presented as a measure of protein abundance on the figures. The data analysis such as imputation, filtering, differential protein expression, differential phosphorylation, and differential protein methylation was carried out on Perseus^22^ and the batch correction was carried out using ComBat on R. Reactome and GO “Biological process” were used for pathway enrichment analysis. Figures were made using ClueGO^23^ on Cytoscape, Clustvis,^24^ and BioRender.

### Phosphoproteome Profiling of DMG Cells Using DIA-MS

We performed comprehensive total proteome and phosphoproteome profiling of 3 DMG cell lines: SU-DIPGXVII, SU-DIPG25, and SU-DIPG50, 3 replicate each, utilizing data-independent acquisition (DIA-MS) approach on the timsTOF Pro platform (Bruker). Protein extraction was carried out as mentioned in the protein extraction method, and peptides were digested with trypsin. A pooled sample comprising equal aliquots from all replicates of all 3 cell lines was used to create a DIA spectral library, generated via data-dependent acquisition on the same instrument. The DIA library creation and spectral annotation were performed using Bruker’s ProteoScape and downstream data analysis was performed using Spectronaut software. Individual samples were run in triplicate using a trapped ion mobility spectrometry-enabled acquisition mode to ensure high reproducibility and sensitivity. The resulting DIA-MS data were searched against the pooled spectral library to identify and quantify phosphopeptides and proteins. Data processing included normalization, filtering for a FDR of <1%, and annotation of phosphorylation sites.

### Kinase Enrichment Analysis and Non-histone Methyltransferase Enrichment Analysis

The list of differentially phosphorylated peptides (phospho-site, peptide sequence, FC, and *P* value) in DMG samples were used for kinase substrates enrichment analysis (KSEA) using KSEA-App as described in Wiredja et al 2017.^25^ The list of active kinases in each DMG sample in our cohort based on KSEA score was used for further analysis. Over-representation analysis of kinases was conducted using KEA3 as described in Kuleshov et al. 2021.^26^ The consensus list of kinases from KSEA and KEA3 was used to generate heatmap. The list of methyltransferase proteins was retrieved from Uniprot human protein database (https://www.uniprot.org/), with the exclusion of annotated histone methyltransferases, and merged with a list of significantly differentially expressed RNA and proteins in DMG to identify enriched non-histone methyltransferases in DMG. The list of enriched non-histone methyltransferases was used to create STRING network (https://string-db.org/) and the RNA/protein expression values were added to the figure on Cytoscape.^27^

### Methyltransferase Assay

Methyltransferase assays were conducted using MTase-Glo™ Methyltransferase Assay (Promega). Purified substrate, recombinant EEF1A1 protein and enzymes, recombinant METTL13 protein, and recombinant METTL21B protein were purchased from Active Motif®. Methyltransferase assay was conducted according to the MTase-Glo™ Methyltransferase Assay manual (Promega).

### Cell Culture and Lentivirus Transduction

All cells were regularly confirmed free of mycoplasma and fingerprinted to confirm their identity. DMG cells were maintained in equal ratios of Neurobasal-A and DMEM/F12 media (Invitrogen) supplemented with 10 mM HEPES, 1 mM sodium pyruvate, 100 μM NEAA, 1 × GlutaMAX-I, 1 × antibiotic/antimycotic, 1 × vitamin A-free B-27 supplement (all from Invitrogen); 20 ng/mL EGF, 20 ng/mL FGF-basic 154, 10 ng/mL PDGF-AA, 10 ng/mL PDGF-BB (all from Shenandoah Biotech); 2 μg/mL heparin (StemCell Technologies). NHA cells were maintained in DMEM (VWR) supplemented with 10% FBS (Wisent). SU-DIPG-XVII and SU-DIPG36 cells were a generous gift from Michele Monje (Stanford University).^28^ PRKACA shRNAs (TRCN0000233527 and TRCN0000356094), PAK2 ShRNAs (TRCN0000002115 and TRCN0000194671), METTL13 ShRNAs (TRCN0000142045 and TRCN0000141891), METTL21B ShRNAs (TRCN0000122880 and TRCN0000138969) or control shRNA (SCH002) plasmids were purchased from Sigma. Lentiviral particles were precipitated with Lenti-X Concentrator (Clontech) and resuspended in Optimem (Invitrogen). Growth curves were generated using trypan blue exclusion assay and Vi-CellXR (Beckman Coulter).

### Immunoprecipitation (IP)

Total protein lysates from tumors and normal brains, which were used for total proteome mass spectrometry analysis, were used for this IP. A total of 500 µL lysates at a concentration of 100 mg/mL were incubated with 2 µL of EEF1A1 antibody overnight with rotation at 4 °C. The next day, 25 µL of protein A/G beads (Thermo Scientific) previously equilibrated in lysis buffer were added for 3 hours and washed 3 times with lysis buffer and resuspended in 100 µL lysis buffer. 10 µL from each sample was used for Western blot analysis.

### Western Blot Analysis

The same tissue protein lysates prepared for profiling total proteome experiments was used for Western blotting. For DMG cell lines, whole cell lysates were prepared in 2× SDS lysis buffer (20 mM Tris [pH 7.4], 20 mM EDTA, 2% SDS, 20% glycerol) and concentration determined by Pierce^TM^ BCA Protein Assay (ThermoScientific). A total of 10 μg of protein was resolved on 10%–20% SDS-PAGE and transferred to PVDF membranes that were blocked and incubated with antibodies diluted in 3% BSA in TBS-T. Binding was detected with enhanced chemiluminescence (Pierce).

### RNA Sequencing

RNAseq data were analyzed from previously published studies in our cohort,^19–21^ as described in the data availability section. For new samples, total RNA was extracted with RNeasy kit (Qiagen) and assessed for quality using Bioanalyzer 2100 (Agilent). TruSeq Stranded Total RNA Library Prep with Ribo-Zero Gold (Illumina) was used for library preparation, followed by sequencing on Illumina HiSeq 2500 (paired-end 150 bp reads) at the Hospital for Sick Children (Toronto). Reads were trimmed with Trimmomatic-v0.3269,^29^ aligned to GRCh37-v75 (human) using STAR v2.5.070 in 2-pass mode,^30^ and duplicate reads were marked with Picard-v2.5.0. Gene expression was quantified with HTSeq, and differential expression was calculated with edgeR, using an absolute fold-change >1 and a Benjamini-Hochberg adjusted *P*-value < .05 as the criteria for differential expression.

### DNA Methylation

Comprehensive methylation profiling of 39 DMG samples at the Microarray Center (University Health Network) using the Illumina Infunium450K or 850K array (Illumina). The previously published DNA methylation in our cohort^19^ is available in GEO. For the new samples, DNA was extracted using the DNeasy kit (Qiagen) according to the manufacturer’s protocols. Bisulphite conversion was conducted using the EZ DNA Methylation Kit (Zymo Research) as per manufacturer’s specifications.

### TePhe Labeling and CyTOF Experiments

DMG cells and Normal Human Astrocytes (NHA) cells were grown in TSM/DMEM media with respective growth factors or 10%FBS for 48h. TePhe, which were synthesized in the Nitz lab, were added to the media 24 hours to a final concentration of 12.5 μM 24 hours before harvesting the cells. The TePhe labeled cells were harvested by centrifugation at 2000 × *g* for 3 minutes and washed twice with PBS. The cells were then incubated with 0.1-μM Cell-ID Ir intercalator in 1.6% FA for 1 hour at room temperature and washed once with PBS and once with dH2O. All cells were analyzed on the Fluidigm Helios system. Each sample was made up of deionized water (1 mL) with 1× EQ Four Element Calibration Beads (Fluidigm). Data was analyzed using FlowJo (Tree Star Inc.) software. The histograms were made using Cytobank community platform.

### Mouse Xenografts

Six-week-old NSG (NOD scid gamma) mice were injected with patient-derived SU-DIPG36 cells carrying scrambled shRNAs or METTL13-shRNAs (10 mice per arm, 5 males and 5 females). Each mouse was injected with 1 μL cell suspension (50 000 cells/μL) into the pontine tegmentum. The injection site was located 0.8 mm anterior and 0.8 mm lateral from the lambda (XY coordinates) and at a depth of 5 mm from the inner base of the skull. Bioluminescence imaging was carried out 2 weeks post-injection to confirm the presence of tumor cells. Mice were monitored daily and euthanized at the Humane endpoint. All animal protocols were approved by the Hospital for Sick Children Institutional Animal Care Committee.

### Statistical Analysis

A 2-way ANOVA was used to determine statistical significance between treatments including drugs or shRNAs for all in vitro cell culture experiments. One-way ANOVA was used to determine the statistical significance of CyTOF experiments. Two-tailed unpaired *t*-test with multiple hypothesis correction with a Benjamini-Hochberg (BH) adjustment was used to determine the significance of the proteomics data. Protein expressions/modifications with a BH-adjusted *P* value < .05 were determined to be statistically significant. Proteins/PTMs with a log2 fold change >1 were considered upregulated and proteins/PTMs with a log2 fold change <−1 were considered downregulated. Pathways with a BH-adjusted *P* value of <.05 were considered significantly enriched. Protein level statistics are included in the mass spectrometry method section. A 2-tailed unpaired *t*-test with Welch’s correction was used to determine the significance of the global protein methylation and phosphorylation in 2 unequal groups of normal and tumor samples. Paired *t*-test, assuming equal variance between samples, was used for methyltransferase assays in vitro. Survival analyses in vivo experiments were evaluated using the log-rank test. Unless otherwise stated, all *P* values were calculated by unpaired *t*-tests, not assuming equal variance between samples. Multiple hypothesis correction (*P*, *P*adj values) was calculated using the BH procedure.

## Results

### Multi-omic Profiling of DMG

**Figure 1.**
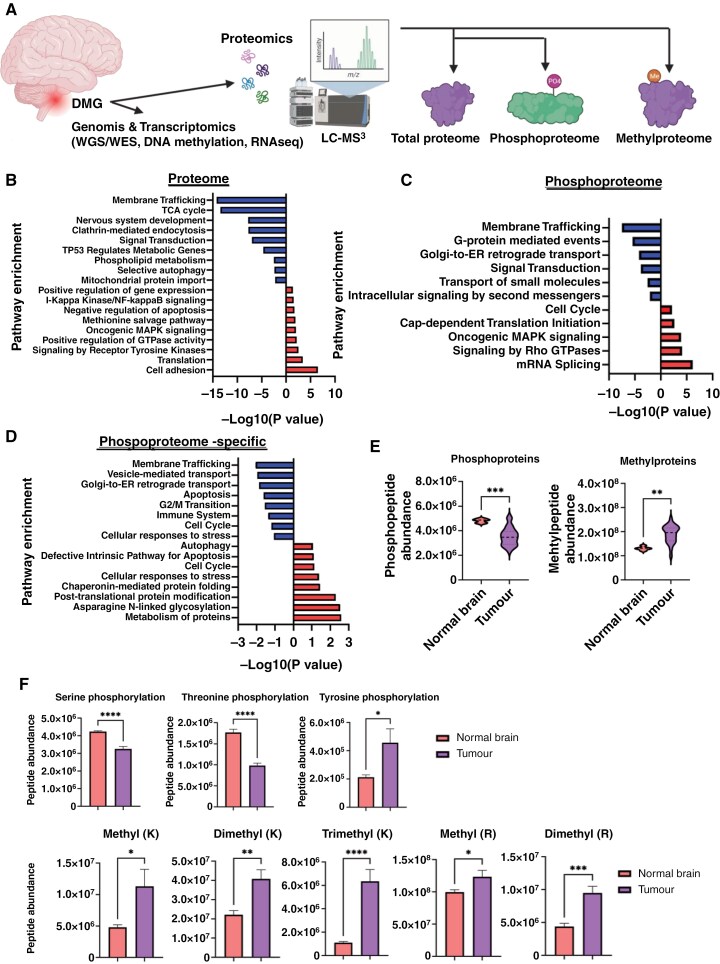
Multi-omic profiling of DMG patient samples. (A) Workflow of multi-omics profiling of DMG samples, including WGS/WES, DNA methylation, bulk RNAseq, total proteome, phosphoproteome, and methylproteome. (B) Pathway enrichment of differentially expressed proteins in DMG. (C) Pathway enrichment of differentially phosphorylated proteins in DMG. (D) Pathway enrichment of differentially phosphorylated proteins that are not differentially expressed in DMG. (E) Global protein phosphorylation and global protein methylation in normal brain and DMG tissues. **P = .001;***P = .0007. (F) Global phosphorylation of serine, threonine, and tyrosine in DMG, and global methylation of lysine and arginine and their each modification in DIPG. *P < .05; **P < .005; ***P < .0005; ****P < .0001.

We performed deep total proteomics of 55 patient samples (39 DMGs and 16 normal brains) using liquid chromatography-tandem mass spectrometry (LC-MS^3^) with matching total RNA sequencing, WES, WGS, and DNA methylation analyses (Figure 1a, Supplementary Table 1). Phosphoproteome and methylproteome were profiled for 30 patient samples. Our cohort consisted of 21 females and 18 males with a median age at diagnosis of 6.8 years and a median survival of 0.8 years (Supplementary Figure 1A, clinical details of the cohort are provided in Supplementary Table 1).

DMG samples were clearly distinct at the transcriptomic, proteomic, and PTM level from normal brain using principal component analysis (Supplementary Figure 1b). We identified 3152 (33% of 9530 total proteins identified) differentially expressed proteins and 7880 (22% of 35 722 RNAs identified) differentially expressed RNAs (coding and non-coding) between DMG and normal brain (Supplementary Tables 2 and 3). Pathway enrichment analysis of differentially expressed proteins in DMG showed enrichment of cell adhesion, translation, GTPase activity, and oncogenic MAPK signaling as well as negative enrichment for membrane trafficking, TCA cycle, and nervous system development (Figure 1B).

mRNA and protein correlations play a vital role in understanding gene expression regulation and its impact on cellular function. The correlation between mRNA and protein levels is generally found to be moderate, ranging from 0.3 to 0.6 in complex samples.^31–34^ However, differentially expressed mRNAs exhibit a significantly better correlation with proteins than non-differentially expressed mRNAs.^35^ In keeping with this, there was moderate to good correlation between protein and mRNA levels of 3059 differentially expressed DMG matched protein:mRNA pairs (based on Uniprot accession to Ensembl ID mapping, excluding transcript variants; Pearson correlation = 0.72, R^2^ = 0.52, Supplementary Figure 1c). However, 395 significantly upregulated proteins were not differentially expressed at the mRNA level and thus would have been missed in transcriptomic studies (Supplementary Tables 2 and 3). These proteins have major functions including the metabolism of RNA, proteins, and vitamins as well as epigenetic regulation, DNA repair, and deubiquitination (Supplementary Figure 1d). This discordance can arise from post-translational regulation including PTMs, protein degradation, and translational efficiency, which all influence protein levels independently of mRNA abundance.^32,36,37^ Proteomics thus provides an orthogonal and more proximal understanding of cellular processes, allowing us to uncover important regulatory mechanisms that would otherwise be missed by mRNA analysis alone.

### PRKACA, PAK2, and AKT1 are Key Drivers of the DMG Phosphoproteome

A total of 218 proteins (47% of 448 identified phosphoproteins) were differentially phosphorylated in DMGs compared to normal brains, with 102 proteins exhibiting higher phosphorylation and 116 proteins showing reduced phosphorylation (Figure 1C, Supplementary Table 4). Pathway analysis of differentially phosphorylated proteins in DMG showed positive enrichment (hyper-phosphorylated proteins in tumors) of mRNA splicing, Rho GTPase signaling, MAPK signaling, cell cycle, and translation initiation and negative enrichment of membrane trafficking, signal transduction, and transport of small molecules (Figure 1C). Proteins that are under-phosphorylated proteins in tumors, such as MADD, DCTN1, and SLC4A10, activate pathways or favor tumorigenesis when unphosphorylated^38–40^ (Supplementary Table 4).

Out of 218 differentially phosphorylated proteins, 80 were not differentially expressed in DMG suggesting that these were only detectable by looking at PTMs, not expressions (Supplementary Table 5). The PTM-enriched proteins, which are involved in protein folding, metabolism of proteins, glycosylation, and autophagy pathways, were not enriched at their expression levels (proteins or mRNAs; Figure 1D).

Quantitative analysis of phosphoproteins in DMG showed that mean global protein phosphorylation was significantly lower in DMG compared to normal brains (*P* = .0007, Figure 1E). This was driven by reduced serine and threonine phosphorylation while, in contrast, tyrosine phosphorylation levels, including oncogenic phosphopeptides, were significantly higher (Figure 1F). While increased global tyrosine phosphorylation has previously been reported in cancer,^41–44^ global loss of serine/threonine phosphorylation in cancer patient tissues has not previously been reported. A total of 29 phosphatases were significantly over-expressed at the protein level in DMG compared to normal brains (Supplementary Table 6). While the subunits of tumor suppressor phosphatases PP2A, PPP2R5E, and PPP2CB (whose substrate VIM was highly phosphorylated in DMG) were downregulated in DMG, oncogenic phosphatases that regulate cell division and DNA repair such as PPP6R1 and PPP4R1 were upregulated in DMG (Supplementary Tables 4 and 6) DEPOD: the human DEphOsphorylation Database.^45^; The observed decrease in phosphorylation levels is primarily attributed to tumorigenesis, especially proteins involved in membrane trafficking and signal transduction pathways (Figure 1C). For example, the diminished phosphorylation of certain proteins, exemplified by DCTN1 involved in lysosomal trafficking and autophagy, serves to activate their functional activity.^38^ Similarly, MADD proteins upon phosphorylation, interact with TRAIL to induce apoptosis; therefore, the negative regulation of MADD through dephosphorylation adds to oncogenesis.^39^ Additionally, dephosphorylation contributes to the stabilization of SLC4 proteins, specifically small molecule transporters. Notably, SLC4A10, a member of this protein family, exhibited significantly reduced phosphorylation in DMG tissues compared to normal brain, suggesting a potential impact on small molecule transport processes (Supplementary Table 4).^40^

**Figure 2.**
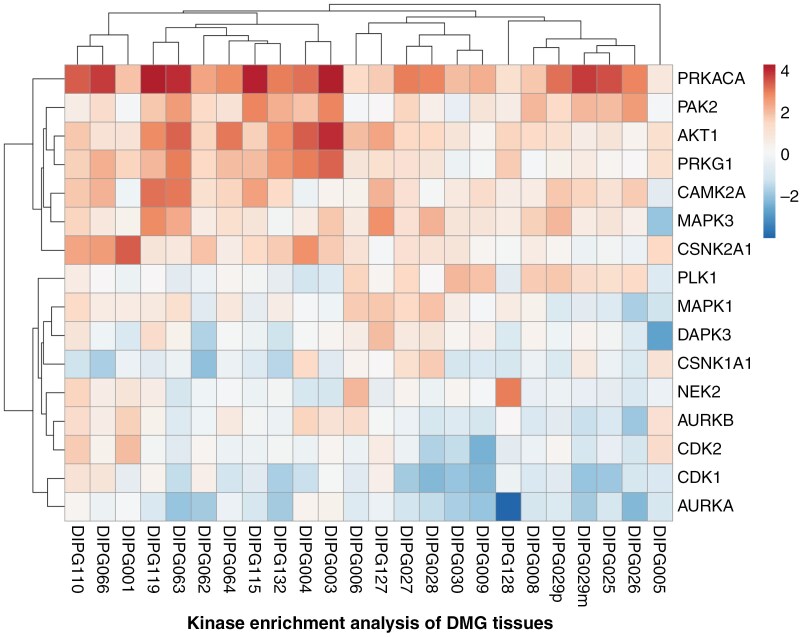
Protein phosphorylation in DMG. Top protein kinases identified by kinase enrichment analysis based on their substrates’ phosphorylation in DMG tissues.

To uncover key drivers of the DMG phosphoproteome, we conducted kinase-substrate enrichment analysis (KSEA).^25^ KSEA enrichment scores were calculated for each kinase based on the fold change of differentially phosphorylated S/T/Y amino acid on the specific peptide sequence of its substrates. KSEA identified PRKACA, PAK2, and AKT1 as the top kinases responsible for differentially phosphorylated proteins in DMG (Figure 2A). To further validate the key drivers of the DMG phosphoproteome, we performed comprehensive phosphoproteome profiling of 3 DMG cell lines (SU-DIPGXVII, SU-DIPG25, and SU-DIPG50), identifying 1323 unique phosphopeptides corresponding to 600 unique phosphoproteins (Supplementary Table 7). To explore signaling pathways driving phosphorylation in DMG cells, we conducted kinase enrichment analysis using KEA3.^26^ This analysis revealed a total of 51 significantly enriched kinases, including key regulators such as CDK1, CDK2, PAK families, AKT1, PRKACA, mTOR, and AURKA (Supplementary Figure 2b). Cell cycle-regulated phosphoproteins such as CDK1 and CDK2 are highly regulated and rapidly dephosphorylated and were likely thus missed in the human samples; however, substrates of AKT1 and PRKACA, which were highly phosphorylated in DMG tumor tissues, also exhibited high phosphorylation levels in the DMG cell lines, validating our tumor findings.

**Figure 3.**
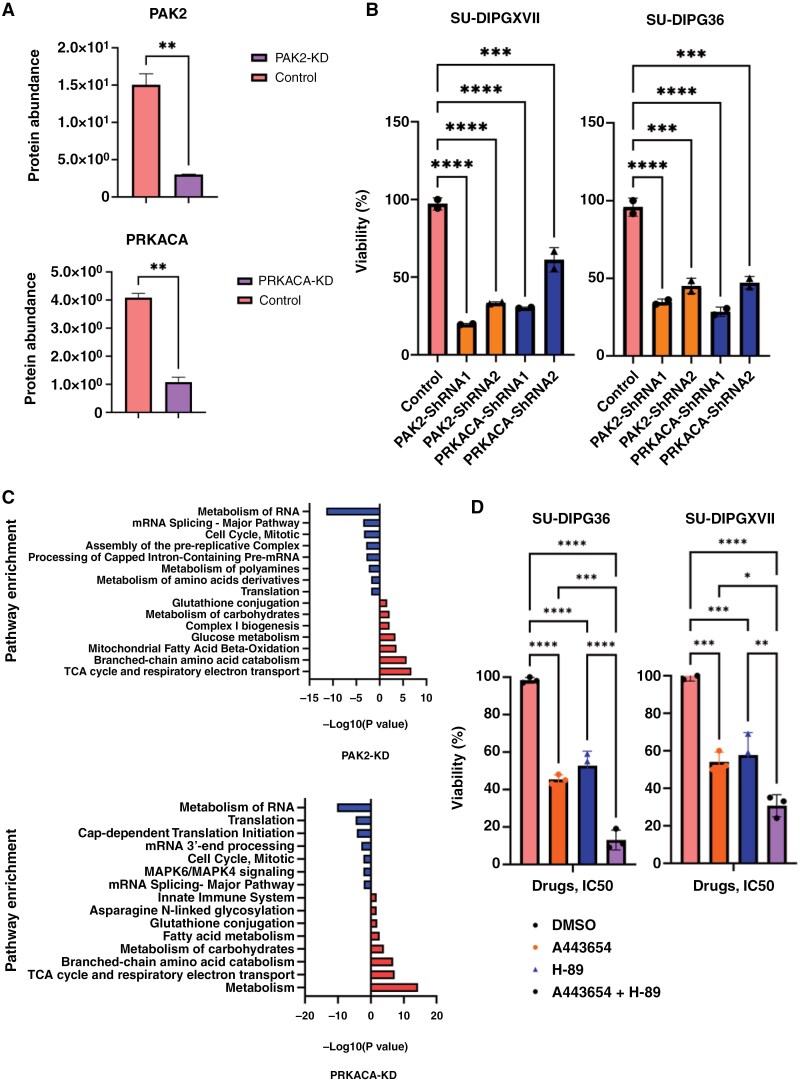
Impact of protein kinases candidates in DMG. (A) Mass spectrometry confirmation of shRNA-based knockdown of PAK2 and PRKACA in DMG (SU-DIPGXVII) cells (ShRNA1&2 combined). **P < .05. (B) Effect of PAK2 and PRKACA knockdown on SU-DIPGXVII and SU-DIPG36 cells growth at 96h in vitro. ***P < .001, ****P < .0001. (C) Pathway enrichment of differentially expressed proteins in PAK2 and PRKACA knockdown cells compared to control cells. KD = Knockdown. (D) Effect of PKA and AKT1 drugs on DMG cells growth at 72 hours in vitro. The IC50 of H-89 and A443654 were used for combination strategy in DMG cells.

We next wished to investigate the potential of these kinases as DMG therapeutic targets. AKT1 inhibition has been previously shown to inhibit DMG cell growth^21^; however, the potential of targeting PRKACA and PAK2 is unknown. We knocked down PRKACA and PAK2 using 2 independent shRNAs and confirmed knockdown at the protein level by mass spectrometry and Western blots (Figure 3A, Supplementary Figure 2a and Tables 9 and 10). Knockdown of PRKACA and PAK2 proteins significantly reduced SU-DIPGXVII and SU-DIPG36 cell growth (Figure 3B). We then used mass spectrometry to identify differentially expressed proteins in PRKACA and PAK2 knockdown SU-DIPGXVII cells and compared them to control scrambled-ShRNAs (Supplementary Tables 9 and 10). Pathway enrichment analysis highlighted translation and cell cycle as the top pathways that were significantly affected in the PRKACA and PAK2 knockdown cells (Figure 3C). DMG single-cell RNAseq data showed PRKACA, and AKT1 were expressed preferentially in malignant cells while PAK2 was also present in the immune and oligodendrocytic populations (Supplementary Figure 2c) suggesting targeting PAK2 can lead to off-tumor toxicity but PRKACA and AKT1 may be more selective targets. AKT1, a key effector of the PI3K/AKT/mTOR pathway, which is frequently dysregulated in DMG and implicated in cell growth, survival, and resistance to therapy. Using CRISPR/Cas9 loss-of-function gene deletion screens, Duchatel et al.^46^ demonstrated that PI3K is a genomic dependency in DMG and that it can be therapeutically targeted through a combination of PKC inhibition and metabolic rewiring using paxalisib and enzastaurin.^46^ This further corroborates our findings on the role of AKT1 and underscores the importance of integrating PI3K/AKT pathway inhibition in future therapeutic strategies.

PRKACA (PKA, catalytic subunit), a central regulator of cAMP-dependent signaling, controls a wide array of processes, including transcription, metabolism, and apoptosis. PAK2 is an important mediator of cytoskeletal dynamics, cell survival, and migration, and is known to interact with RAS and MAPK pathways.^47^ These pathways are relevant in H3K27M-driven DMGs, where RAS/MAPK signaling supports tumor growth and resistance mechanisms. In DMG, the substrates we identified for PRKACA primarily regulate post-transcriptional regulatory proteins, while those of AKT1 and PAK2 predominantly influence components of the translation initiation complex, alongside other canonical proteins phosphorylated by these kinases.

There is currently no drug that directly targets PRKACA; however, it can be inhibited indirectly by targeting the PKA holoenzyme (Protein Kinase A), of which PRKACA is the catalytic subunit. To explore this, we tested H-89 and A443654, which inhibit PKA and AKT1 activity, respectively, in DMG cells. Both drugs significantly inhibited DMG cell growth at IC50s concordant with other tumors in which they are effective (IC50 values of A443654 were 130 nM and 180 nM in SU-DIPG36 and SU-DIPGXVII, respectively; IC50 values of H-89 were 7 μM and 3.1 μM in SU-DIPG36 and SU-DIPGXVII, respectively [Supplementary Figure 3a]).^21,48,49^ Subsequently, we combined both drugs at their respective IC50 concentrations and observed a significantly greater inhibitory effect on DMG cell growth compared to either drug alone (Figure 3D). These findings align with our shRNA-based knockdown experiments (Figure 3A and B), further confirming the critical role of these kinases in DMG and as viable targets for pharmacological intervention.

### METTL13 and METTL21B are Key Drivers of the DMG Methylproteome

**Figure 4.**
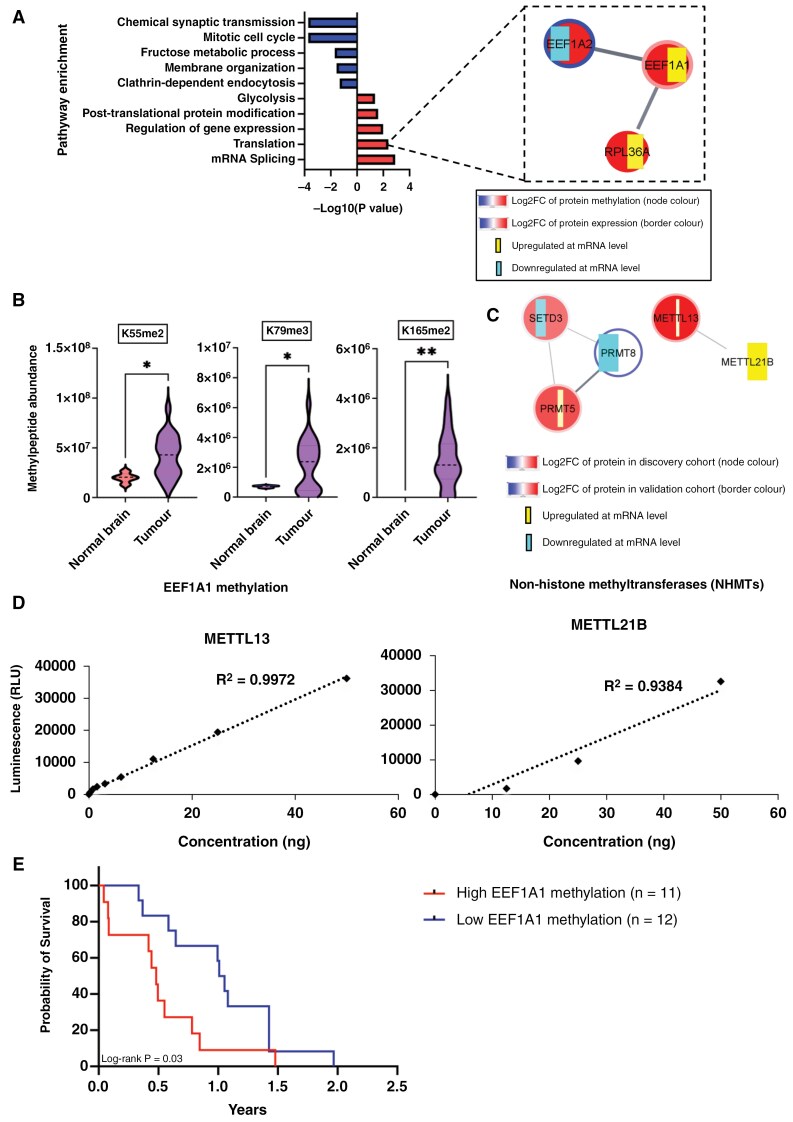
Protein methylation and survival correlation in DMG. (A) Pathway enrichment of differentially methylated proteins in DMG. Translation elongation proteins are highlighted. The width of the RNA heat strip (yellow or blue) varies by Log2FC. (B) Non-histone protein, EFF1A1 is highly methylated at K55, K79, K165 in DMG. (C) Top 5 enriched (mRNA or protein expression) NHMTs in DMG. METTL13, METTL21B, and PRMT5 are upregulated in DMG tissues compared to normal brain. The width of the RNA heat strip (yellow or blue) varies by Log2FC. (D) In vitro methyltransferase assays showing the activity of METTL13 and METTL21B on EEF1A1 methylation. The methyltransferase activity between any 2 concentrations is significant (*P* < 0.05). (E) The Kaplan–Meier survival curve, which represents the relationship between EEF1A1 methylation and survival in DMG patients, revealed a significant association. Median methylation value was used as a cutoff value to separate low versus high.

Global reduction in histone H3K27 trimethylation (H3K27me3) is a hallmark in H3K27-altered DMG^50–53^; however, methylation of non-histone proteins has not been comprehensively studied. To identify global protein methylation, with an emphasis on non-histone protein methylation in DMG and normal brain, we used strong cation exchange (SCX) followed by LC-MS.^3^ We found that global protein methylation was significantly (*P* = .01) higher in DMG tissues compared to normal brain tissues (Figure 1E). Dimethyl-K, dimethyl-R, and trimethyl-K were most significantly enriched in DMG tissues (Figure 1F). There is a clear clustering between DMGs and normal brain samples based on their methylproteome profiles (Supplementary Figure 1b). This grouping appears to be general, as no distinct differences were observed among DMG samples with different H3 mutation statuses. We identified 38 (23% out of 166 captured non-histone methylproteins) significantly differentially methylated proteins, with 20 proteins hyper-methylated and 18 proteins under-methylated in DMG tissues compared to normal brain (Supplementary Table 8). These differentially methylated proteins were associated with upregulation of translation (eg, EEF1A1 and EEF1A2), and splicing (eg, HNRNPA1, HNRNPAB, and HNRNPA2B1; Figure 4A). Splicing was found to be a non-mutagenic mechanism that activates oncogenic pathways in glioma^20^ and much of this machinery is regulated through protein methylation.^54,55^ While GFAP was the most methylated protein, the translation elongation protein EEF1A1 was the most highly methylated translation machinery protein in DMG (Figure 4A, Supplementary Table 8). Trimethylation at K79 and dimethylation at K55 and K165 of EEF1A1 were significantly higher in DMG tissues compared to normal brains (Figure 4B).

To uncover regulators of the DMG methylome, we took advantage of our multi-omic data to query non-histone methyltransferase expression at the mRNA and protein levels in DMG. METTL13, METTL21B, and PRMT5 were the most significantly overexpressed (Figure 4C). Single-cell RNAseq data confirmed that *METTL13*, *METTL21B,* and *PRMT5* were enriched in malignant cells in DMG (Supplementary Figure 2c).

PRMT5 is an arginine methyltransferase while METTL13 and METTL21B are METTL family lysine methyltransferases that methylate EEF1A1,^56–58^ one of the most highly methylated proteins in DMG. We confirmed the methyltransferase ability of METTL13 and METTL21B on EEF1A1 proteins in vitro (Figure 4D). Interestingly, in our cohort high EEF1A1 methylation was associated with poor prognosis in DMG suggesting it may be contributing to more aggressive behavior (Figure 4E). EEF1A1 is ubiquitously expressed and is one of the most abundant proteins in cells.^59^ Although EEF1A1 is an essential protein and thus not itself a viable therapeutic target, its methylation by METTL13 and METTL21B is not an essential modification and is potentially targetable. Importantly, high EEF1A1 methylation is required by cancer cells to meet their translational demand^57^ and hence, METTL13 and METTL21B are essential proteins in cancer cells but not normal cells. Indeed, CRISPR knockout studies have demonstrated that *METTL13* was not an essential gene in normal cell lines but more likely to be essential in osteosarcoma, diffuse glioma, non-Hodgkin lymphoma, melanoma, and liposarcoma cell lines (DepMap: Cancer dependency map^60,61^). The perturbation gene effect score of METTL13 was considerably lower in cancer cells than in normal cell lines suggesting that targeting this methyltransferase may have limited toxicity in normal tissues (Supplementary Figure 4a).

**Figure 5.**
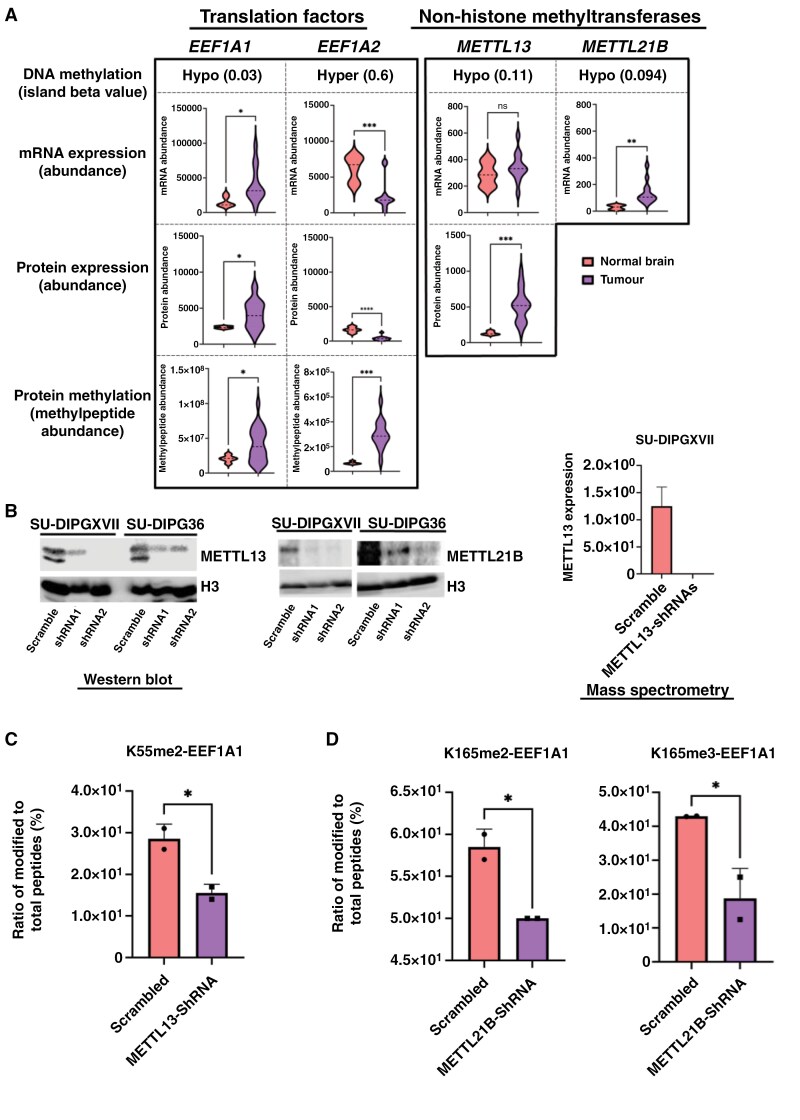
Effect of methyltransferase candidates, METTL13 and METTL21B in DMG cells. (A) Multi-omics enrichment of EEF1A1, EEF1A2, METTL13, and METTL21B in DMG. METTL21B is a missing value in proteome data. *P < .05; **P < .005; ***P < .0005; ****P < .0001; ns-not significant. (B) Western blot and mass spectrometry confirmation of shRNA-based knockdown of METTL13 and METTL21B in DMG cells. METTL21B (missing value) was not capturable in MS, shRNA1 and shRNA2 were combined for the METTL13 MS analysis. (C) The knockdown of METTL13 and METTL21B significantly reduced methylation on K55 of EEF1A1 in SU-DIPG36 cells. *P < .05. ShRNA1 and ShRNA2 were combined for the analysis. (D) The knockdown of METTL21B significantly reduced methylation of K165 of EEF1A1 in SU-DIPG36 cells. *P < .05. ShRNA1 and ShRNA2 were combined for the analysis.

Figure 5A shows a multiomics view of DNA methylation, mRNA expression, and protein expression of the METTL13 and METTL21B, and their substrates EEF1A1 and EEF1A2, in DMG. Out of the 2 isoforms, EEF1A1 and EEF1A2, only EEF1A1 was upregulated in DMG compared to normal brain tissues. In contrast, EEF1A2 was expressed at very low levels in the normal brain and was even further downregulated in DMG. METTL13 was highly expressed at the protein level and its substrates were highly methylated, indicating that METTL13 was active in DMG. METTL21B was highly significantly expressed at the mRNA level in DMG but not identified (missing value) in our proteome cohort. Missing values in mass spectrometry-based large proteomics data are quite common and can be addressed by multiple imputation methods or targeted proteomics.^62^ The enriched expression levels of EEF1A1, METTL13, and METTL21B in DMG were confirmed by Western blot analysis (Supplementary Figure 3b). In addition, EEF1A1 methylation in DMG tissues was validated through immunoprecipitation of EEF1A1 from tumor protein lysates, followed by Western blot analysis using anti-pan methyl lysine antibodies (Supplementary Figure 3c). Thus, METTL13 and METTL21B were over-expressed, and their substrates were highly methylated, indicating that these non-histone methyltransferases were active in DMG and may represent a novel therapeutic target.

### METTL13 Knockdown Decreases Expression of the Oncoproteome and Reduces Cell Viability in DMG

As there are no specific inhibitors available to study the effects of METTL13 and METTL21B inhibition in DMG cells, we used 2 independent shRNAs for each gene to reduce the expression of these genes. The knockdown of METTL13 and METTL21B at the protein level was confirmed by Western blot and led to a significant reduction in EEF1A1 protein methylation, specifically of lysine 55 and lysine 165 (Figure 5B, C, and D).

**Figure 6.**
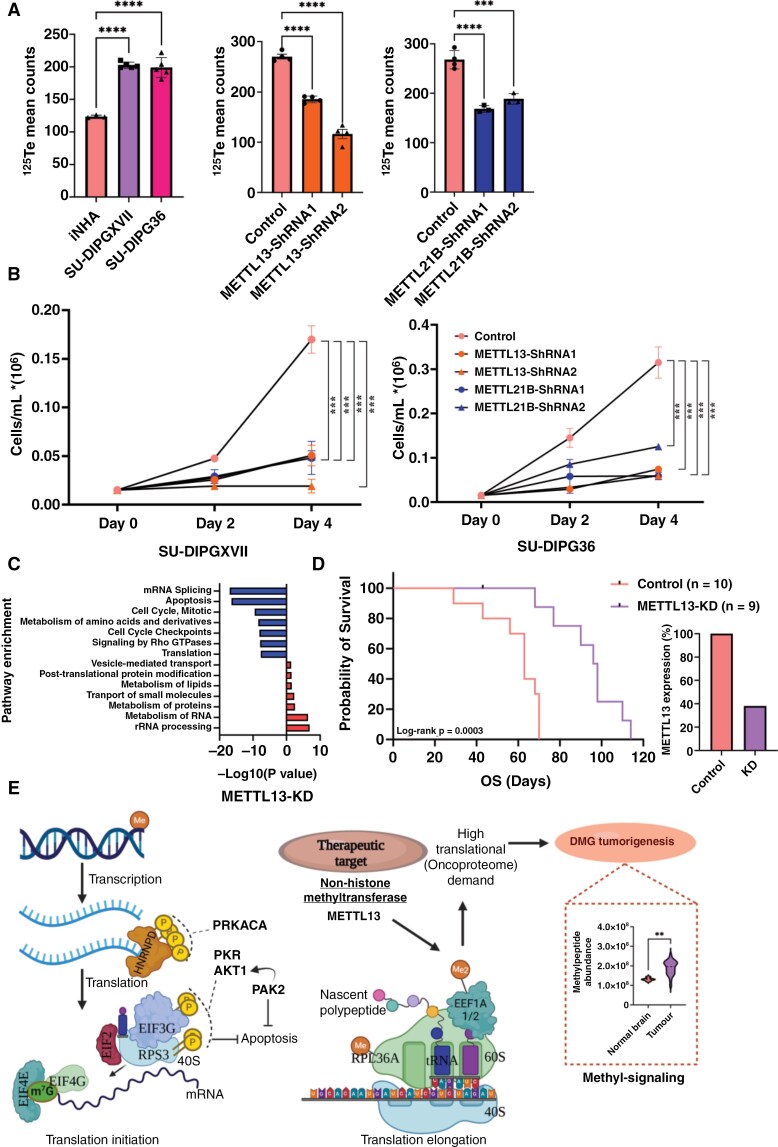
Knockdown of METTL13 and METTL21B reduced global protein synthesis and cell growth in vitro. (A) The knockdown of METTL13 and METTL21B significantly reduced global protein synthesis in SU-DIPG36 cells. DMG cells have significantly higher protein synthesis than control iNHA cells. ***P < .0005; ****P < .0001. Mean ^125^Te counts were retrieved from cells that displayed ^191^Ir signals, after gating. (B) The effect of knockdown of METTL13, and METTL21B on DMG cells growth in vitro. (C) Pathway enrichment of differentially expressed proteins in METTL13 knockdown cells compared to control cells. (D) METTL13 knockdown improves mice survival in DMG xenograft models in vivo (Log-rank *P* = .0003). Median survival days are 63 and 97 in control and METTL13-KD mice, respectively. METTL3-ShRNA2 was used for this experiment. (E) Integrative model of kinases and METTL13 substrates and their impact on translation machinery and DMG tumorigenesis.

DMG has widespread loss of H3K27me3 and more open chromatin creating a permissive environment for transcription.^52,63,64^ With high methylation of translation elongation protein EEF1A1, we hypothesized that DMGs are highly translationally active, with methylated K55 favoring an oncoproteome and cell growth. We used L-2-tellurienylalanine (TePhe) as a noncanonical amino acid for the direct measurement of protein synthesis by mass cytometry without post-experiment labeling.^65,66^ We found significantly (*P* < .0001) elevated protein synthesis rates in the DMG cell lines SU-DIPGXVII and SU-DIPG36 as compared to NHA (Figure 6A, Supplementary Figure 5). METTL13 knockdown and the consequent reduction in EEF1A1 methylation was associated with significantly (*P* < .001) decreased protein synthesis in SU-DIPGXVII and SU-DIPG36 cells and reduced cell growth (Figure 6A and B, Supplementary Figure 5). Additionally, we conducted the METTL13 knockdown in SU-DIPG25 and normal human astrocytes (NHAs, isolated from a DMG patient) and observed that the knockdown did not affect the growth of NHA in vitro (Supplementary Figure 3d) supporting a DMG-specific dependency on METTL13. Interestingly, the decrease in protein synthesis in SU-DIPGXVII and SU-DIPG36 cells was not generalized—rather, the knockdown of METTL13 and decreased K55 methylation of EEF1A1 led to reduced translation of a distinct set of proteins. Previous METTL13-KO studies showed METTL13’s codon bias for lysine (AAA and AAG) and histidine, i.e. proteins with high lysine undergo slower translation rates whereas alanine codons undergo higher translation rates.^58^ In DMG, METTL13 knockdown affected protein synthesis in a similarly specific fashion importantly accompanied by reduced cell growth (Figure 5D and C). The Knockout of METTL13 reduced codon-specific translation rates; downregulated proteins had high lysine and glutamic acid composition (eg, splicing protein SF3A2) and upregulated proteins had high alanine and leucine composition (eg, Serine protease HTRA1) in the METTL13 knockdown cells compared to control cells (Supplementary Figure 6 and Table 11).

Overall, our data suggests that in DMG, METTL13 mediates its oncogenic effects via methylation of EEF1A1, accelerating the translation of proteins enriched in lysine and at the expense of proteins rich in alanine and shaping a DMG-specific oncoproteome (Supplementary Figure 6). Mass spectrometry analysis of differentially expressed proteins in METTL13 knockdown DMG cells, but not METTL21B knockdown DMG cells, highlighted reduced mRNA splicing, cell cycle, metabolism of amino acids, and translation; all important to maintain oncogenesis (Figure 6C, Supplementary Tables 11 and 12). While the METTL21B knockdown showed a general effect on protein synthesis, the METTL13 knockdown showed a shift in oncoproteome synthesis. These findings support the idea that inhibition of the methyltransferase activity of METTL13 will not just reduce protein synthesis in general, but rather target specific oncogenic processes (oncoproteome) supporting DMG survival (Supplementary Figure 6, Figure 6B and C).

Since there are currently no drugs available to target METTL13, we evaluated the effect of METTL13 knockdown in DMG xenograft models in vivo using SU-DIPG36 cells transduced with either scramble shRNAs or METTL13-specific shRNAs. Prior to in vivo experiments, we confirmed METTL13 knockdown in SU-DIPG36 cells via western blot analysis achieving approximately 60% knockdown at the protein level (Supplementary Figure 3e). Mice injected with METTL13-knockdown DMG cells had a significantly longer survival compared to those injected with scramble control DMG cells despite the relatively modest knockdown (median survival 97 versus 63 days, respectively, Log-rank *P* = .0003, Figure 6D). This preclinical validation highlights the therapeutic potential of targeting METTL13 in DMG patients.

## Discussion

DMGs are diffusely infiltrative, aggressive brain cancers which are the main cause of cancer-mediated death in children. The quest for novel therapeutic strategies is spurred by the urgency to confront the unique challenges posed by DMG. Despite significant strides in genomics and transcriptomics, which have unveiled key genetic alterations steering DMG development, a critical gap persists in our understanding and has limited our ability to treat these patients. Multi-omic analysis, integrating proteomics, PTM profiling, DNA methylation, and transcriptomics, offers a powerful approach to unravel the intricate molecular mechanisms driving the pathogenesis of DMG and other cancers. Here we conducted deep, high-resolution profiling of the DMG proteome. This enabled us to detect widespread changes in protein expression, with 33% of identified proteins exhibiting significant differential expression compared to normal brain tissues (Figure 1B, Supplementary Table 2), many of which would not have been detected using genomic and transcriptomic analyses. Significantly, we noted a discrepancy between the mRNA and protein levels of specific targets (Supplementary Figures: 1c and 1d). Within our cohort, 5% and 2% of the total proteome that were captured correspond to phosphoproteins and methylproteins, respectively. Protein methylation constitutes only 3% of all reported PTMs in general PTMCuration.^67^: The presence of detectable PTMs is usually primarily influenced by signaling events rather than total protein amounts.^68^ Our study has pinpointed several novel therapeutic targets for DMG, including kinases and methyltransferases. Notably, METTL13, a non-histone methyltransferase, exhibited significantly increased protein expression in DMG despite not being differentially expressed at the mRNA level (Figure 5A). Similarly, we found the translation initiation factor EIF3G to be highly phosphorylated in DMG without significant upregulation at the protein level (Supplementary Tables 2 and 4). These findings underscore that studying gene regulation at the transcriptional, post-transcriptional, translational, and post-translational level, uniquely leads to a more comprehensive understanding of tumorigenesis.

Our proteomics analysis yielded valuable insights into the intricate patterns of post-transcriptional gene regulation of the splicing and translation pathways by both phosphorylation and methylation (Figures 1C and 4A). Specifically, multiple PTMs converge in DMG to regulate translation, suggesting that the kinases and methyltransferases active in DMG exert their influence on oncogenesis, in part, through the translation machinery (Figure D. These findings highlight the pivotal role of PTMs in shaping the functional protein landscape of DMG, underscoring the significance of PTMs as potential therapeutic targets. In DMG cells, we found that upregulation of the methyltransferase METTL13 leads to high levels of methylation of the translation regulator EEF1A1 which, consistent with previous reports in Ras-driven pancreatic and lung cancer models,^57^ was accompanied by increased rates of protein synthesis. Importantly, we found that elevated EEF1A1 methylation was associated with poor prognosis in DMG (Figure 4E). EEF1A1 is an essential protein and one of the most abundantly expressed cellular proteins, with roles in cell growth and proliferation, cytoskeleton organization, mitotic apparatus formation, and signal transduction.^69–71^ METTL13 and METTL21B methylate EEF1A1 at K55 and K165, respectively.^56,57^ EEF1A1-K55 methylation was found to be a regulator of oncogenic translation, while EEF1A1-K165 methylation exerts a broader influence on translation regulation. Our results indicate that K55 methylation selectively facilitates the translation of a subset of proteins characterized by high lysine and glutamic acid content in DMG cells (Supplementary Figure 6).

Consistent with our findings in DMG, METTL13 upregulation is a general trend in cancer, including breast, colorectal, prostate, lung, and liver tumors (Supplementary Figure 4b), and METTL13 has been shown to act as an oncogenic driver in vivo.^57,72^ Furthermore, amplification of *METTL13* and *METTL21B* was recently uncovered in a subset of cancers, with *METTL13* significantly associated with poor prognosis.^73^ METTL13 is not an essential gene in normal tissues but, based on CRISPR screening from the DepMap project, cancers including diffuse glioma, liposarcoma, colorectal, and prostate adenocarcinoma appear to be dependent on its activity (Supplementary Figure 4a).

METTL13 depletion inhibited cancer cell proliferation and significantly reduced in vivo tumorigenesis of Ras-driven pancreatic and lung cancer mouse models and patient-derived xenografts.^57^ Our findings have highlighted the pivotal role of Ras/MAPK signaling in DMG and H3K27M-driven glioma,^21^ suggesting that METTL13 exerts a comparable effect in DMG. Notably, the loss of METTL13 function altered translation dynamics and resulted in changed translation rates of specific codons, unlike METTL21B (Supplementary Figure 6).^58^ In DMG, METTL13 knockdown affects protein synthesis in a similar specific fashion, disrupting the oncoproteome, leading to cell growth inhibition, and showing survival benefits in mice xenograft models in vivo (Figure 6A, B, C, and D). Hence, targeting METTL13 holds great promise as a therapeutic strategy for DMG patients.

This study presents a comprehensive profile of the DMG proteome, phosphoproteome, and methylproteome, allowing us to develop an integrative model of kinase and methyltransferase activity and their impact on DMG (Figure 6E). It highlighted the regulation of translation machinery proteins identified by our PTM profiling and the role of methyl-signaling by non-histone methyltransferases in EEF1A1 methylation and DMG tumorigenesis. These findings support the notion that targeting the methyltransferases responsible for EEF1A1 methylation is a viable strategy to disrupt protein synthesis in a cancer-specific manner and impede the growth of DMG cells as part of a multi-agent approach to therapy. These discoveries represent a significant step forward in understanding the methyl-signaling mechanisms underpinning DMG tumorigenesis and pave the way for the development of novel and targeted therapies for this devastating disease and other incurable cancers.

### Limitations of the Study

Our study, while impactful, is not without its limitations. Firstly, the inherent constraints of mass spectrometry detection underscore the need for cautious interpretation of negative values, given its potential limitations in detecting low-abundance proteins and PTMs. Secondly, the utilization of autopsy tissues introduces potential loss of phosphoprotein detection associated with postmortem changes. With human tissue, particularly postmortem tissue, it is not possible to snap freeze immediately upon devitalization thus we are certainly missing some phosphoproteins that dephosphorylate rapidly. To account for both of these variables and to ensure that the lack of phosphopeptides in the tumor was not an artifact, we used matched normal tissue for each patient tumor sample where consent allowed. Lack of phosphoproteins in the tumor was only reported if they were lost versus normal brain tissue subject to the same postmortem interval and handling as the tumor. Furthermore, to address potential concerns related to tissue degradation, we employed total intensity normalization strategies to account for global protein abundance differences between samples. Thus, while some phosphopeptides may be missing due to degradation, the differences between tumor and normal are robust. The retained phospho-sites highlight the tumor’s dependency on specific signaling pathways despite the overall decrease in global phosphorylation.

## Supplementary material

Supplementary material is available online at *Neuro-Oncology* (https://academic.oup.com/neuro-oncology).

noaf033_suppl_Supplementary_Tables

noaf033_suppl_Supplementary_Figures

## Data Availability

DNA methylation data are available in GEO under accession number GSE49822. Previously published sequencing data on our cohort^19–21^ are deposited in the European Genomics Archive (EGA); WGS data are available in EGA under accession number EGAS00001000575; WES data are available in EGA under accession numbers EGAS00001000575, EGAD00001008278 and EGAD00001006450; RNA-Seq data are available in EGA under accession number EGAD00001006450, EGAD00001008279. The total proteome and phosphoproteome are available in EGA under accession number EGAS00001007341. The methylproteome data is available in EGA under accession number EGAS00001007342. The data are available under controlled access to comply with data protection regulations and can be accessed by application to the data access committee via C.H. (cynthia.hawkins@sickkids.ca). Custom code was not used in this study. Any modifications of parameters made in the code were mentioned in the respective method section.
