# Supplementary material for: The proteomic landscape of diffuse midline glioma highlights the therapeutic potential of non-histone protein methyltransferases
Source: Neuro Oncol. 2025 Feb 15;27(7):1829–46. doi: 10.1093/neuonc/noaf033 (PMC12417832; doi:10.1093/neuonc/noaf033)
Supplement: noaf033_suppl_Supplementary_Figures [file noaf033_suppl_supplementary_figures.pptx]

## Slide 1
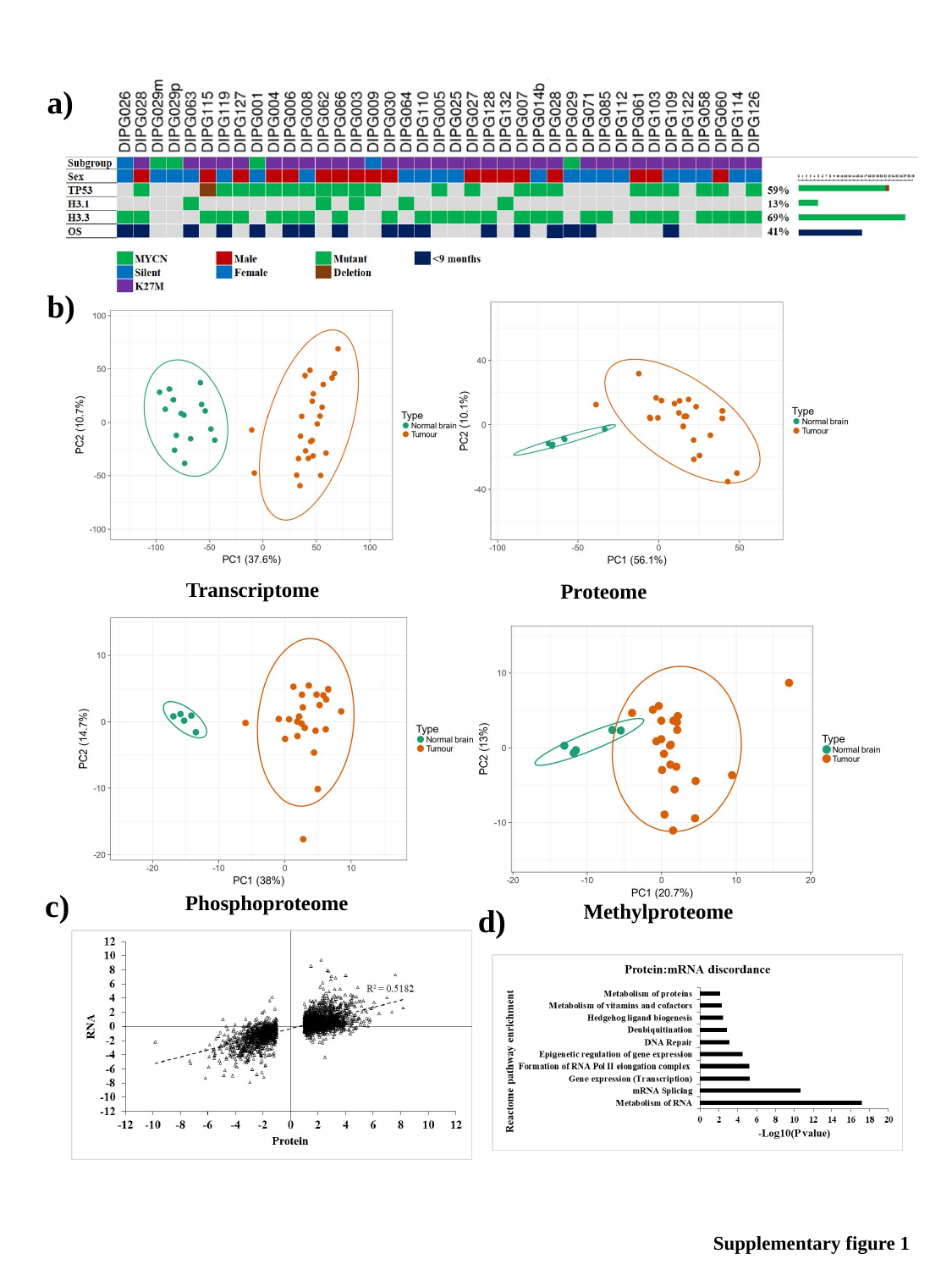

a)
b)
Transcriptome
Proteome
c)
Phosphoproteome
Methylproteome
d)
Supplementary figure 1

## Slide 2
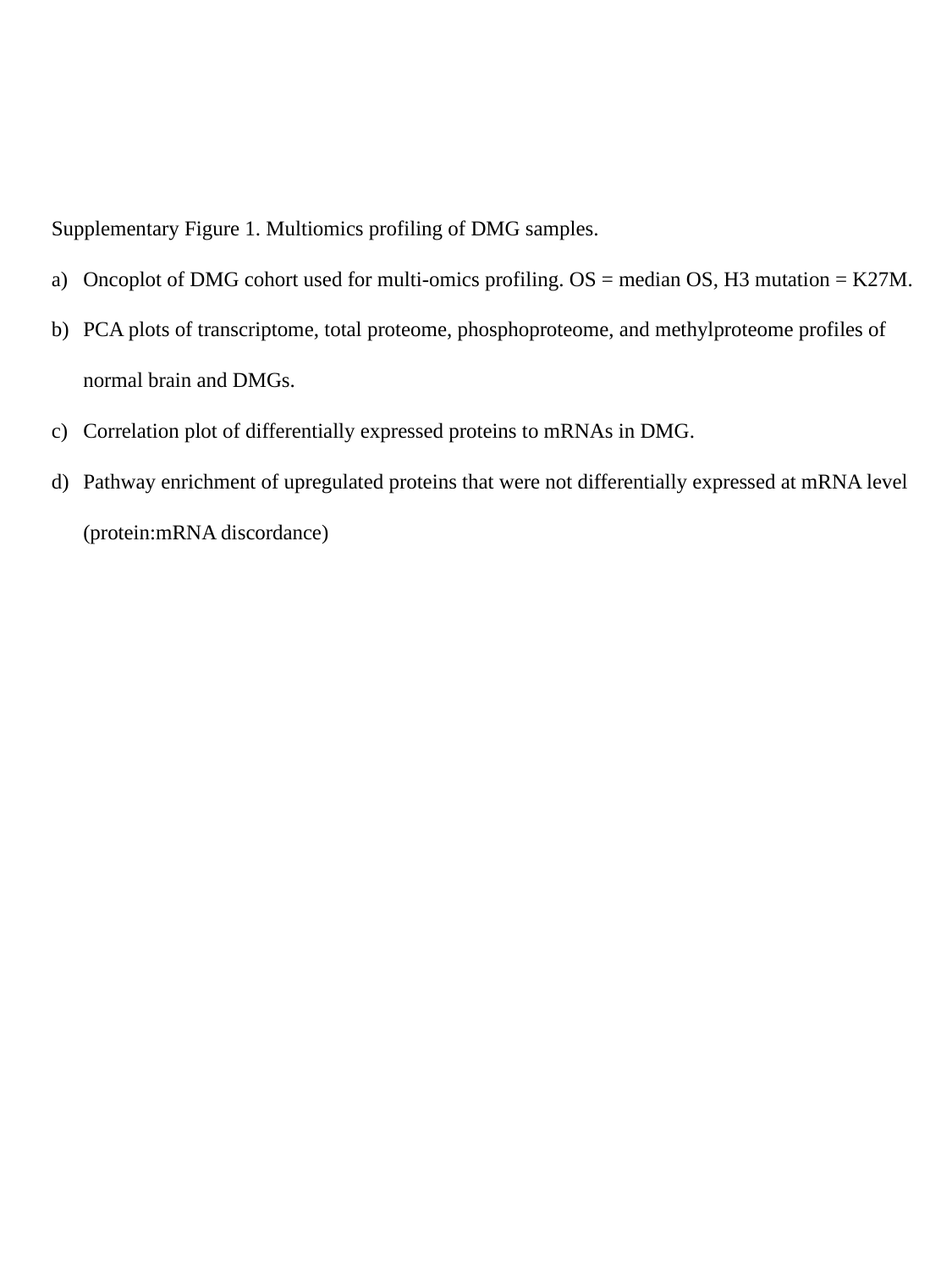

Supplementary Figure 1. Multiomics profiling of DMG samples.
Oncoplot of DMG cohort used for multi-omics profiling. OS = median OS, H3 mutation = K27M.
PCA plots of transcriptome, total proteome, phosphoproteome, and methylproteome profiles of normal brain and DMGs.
Correlation plot of differentially expressed proteins to mRNAs in DMG.
Pathway enrichment of upregulated proteins that were not differentially expressed at mRNA level (protein:mRNA discordance)

## Slide 3
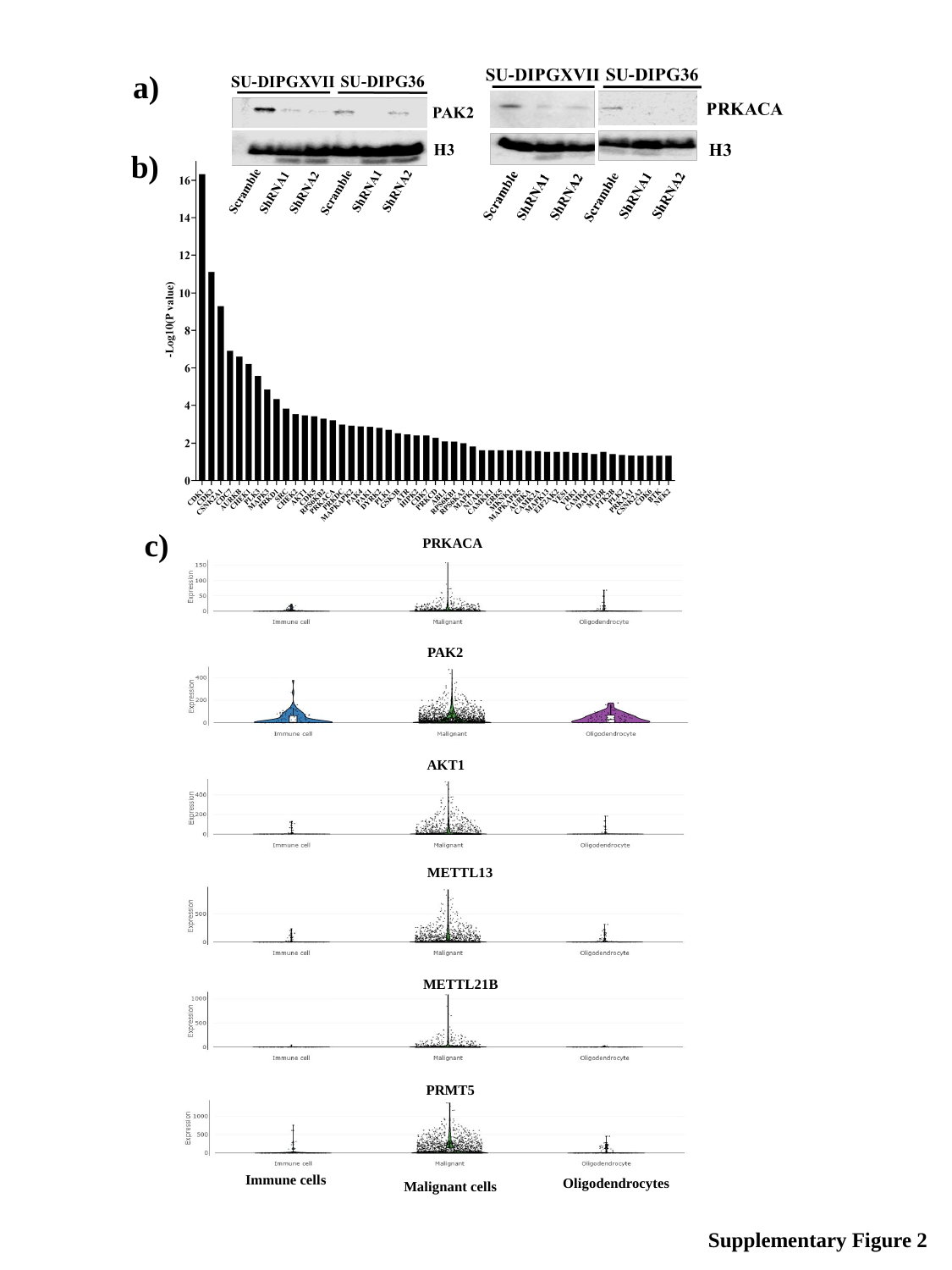

a)
b)
c)
PRKACA
PAK2
AKT1
METTL13
METTL21B
PRMT5
Immune cells
Oligodendrocytes
Malignant cells
Supplementary Figure 2

## Slide 4
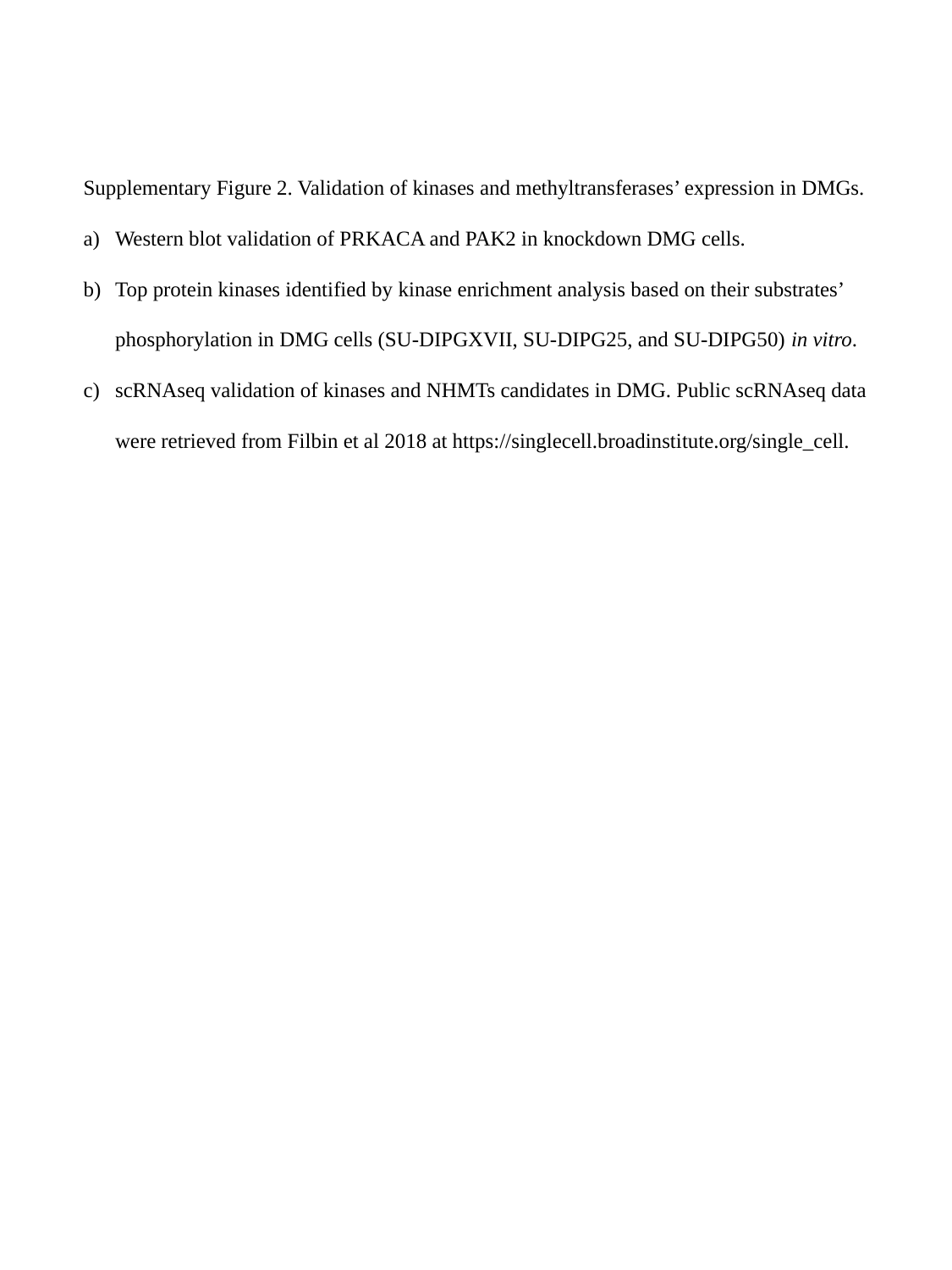

Supplementary Figure 2. Validation of kinases and methyltransferases’ expression in DMGs.
Western blot validation of PRKACA and PAK2 in knockdown DMG cells.
Top protein kinases identified by kinase enrichment analysis based on their substrates’ phosphorylation in DMG cells (SU-DIPGXVII, SU-DIPG25, and SU-DIPG50) in vitro.
scRNAseq validation of kinases and NHMTs candidates in DMG. Public scRNAseq data were retrieved from Filbin et al 2018 at https://singlecell.broadinstitute.org/single_cell.

## Slide 5
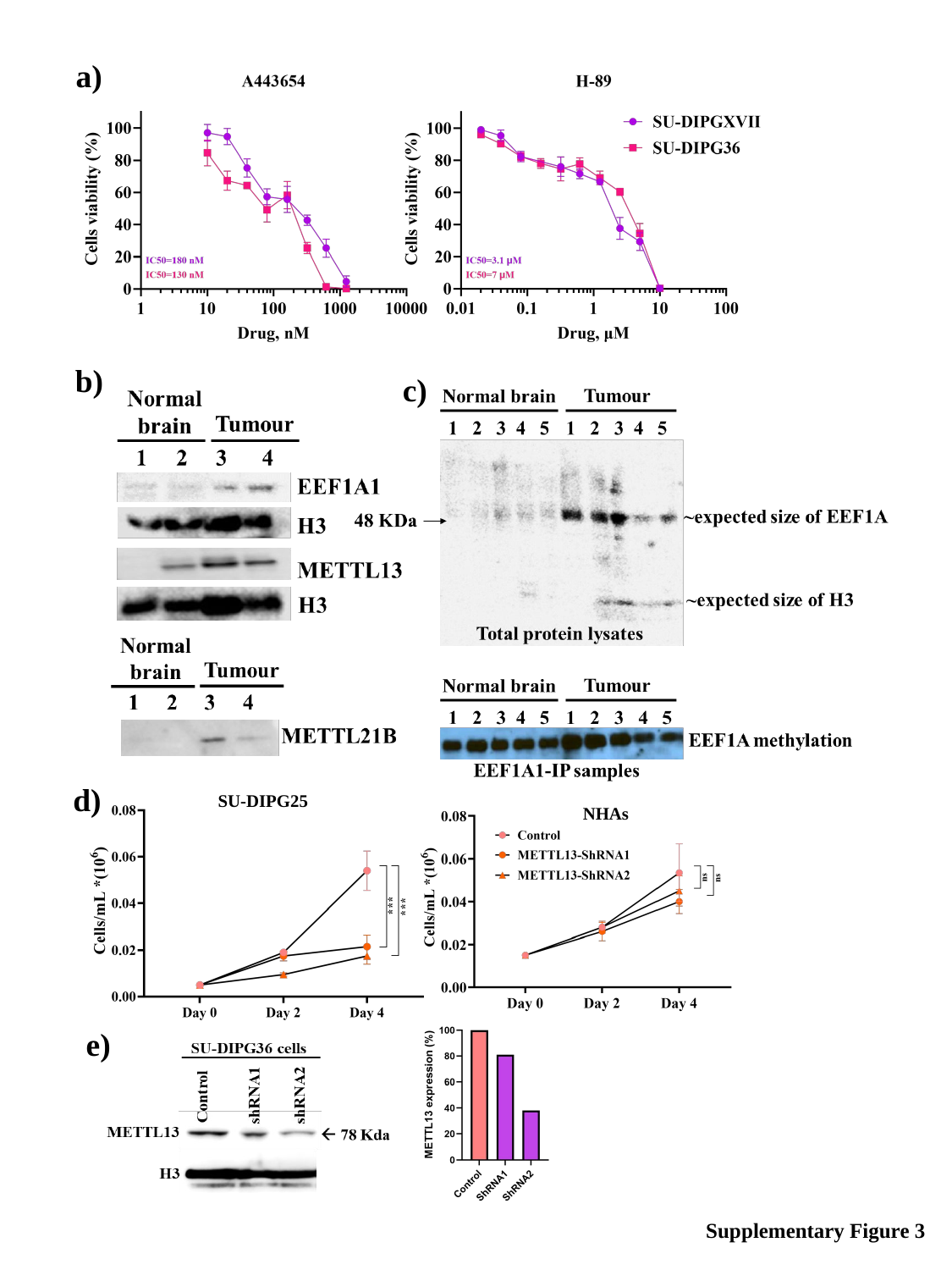

a)
b)
c)
d)
SU-DIPG25
NHAs
e)
Supplementary Figure 3

## Slide 6
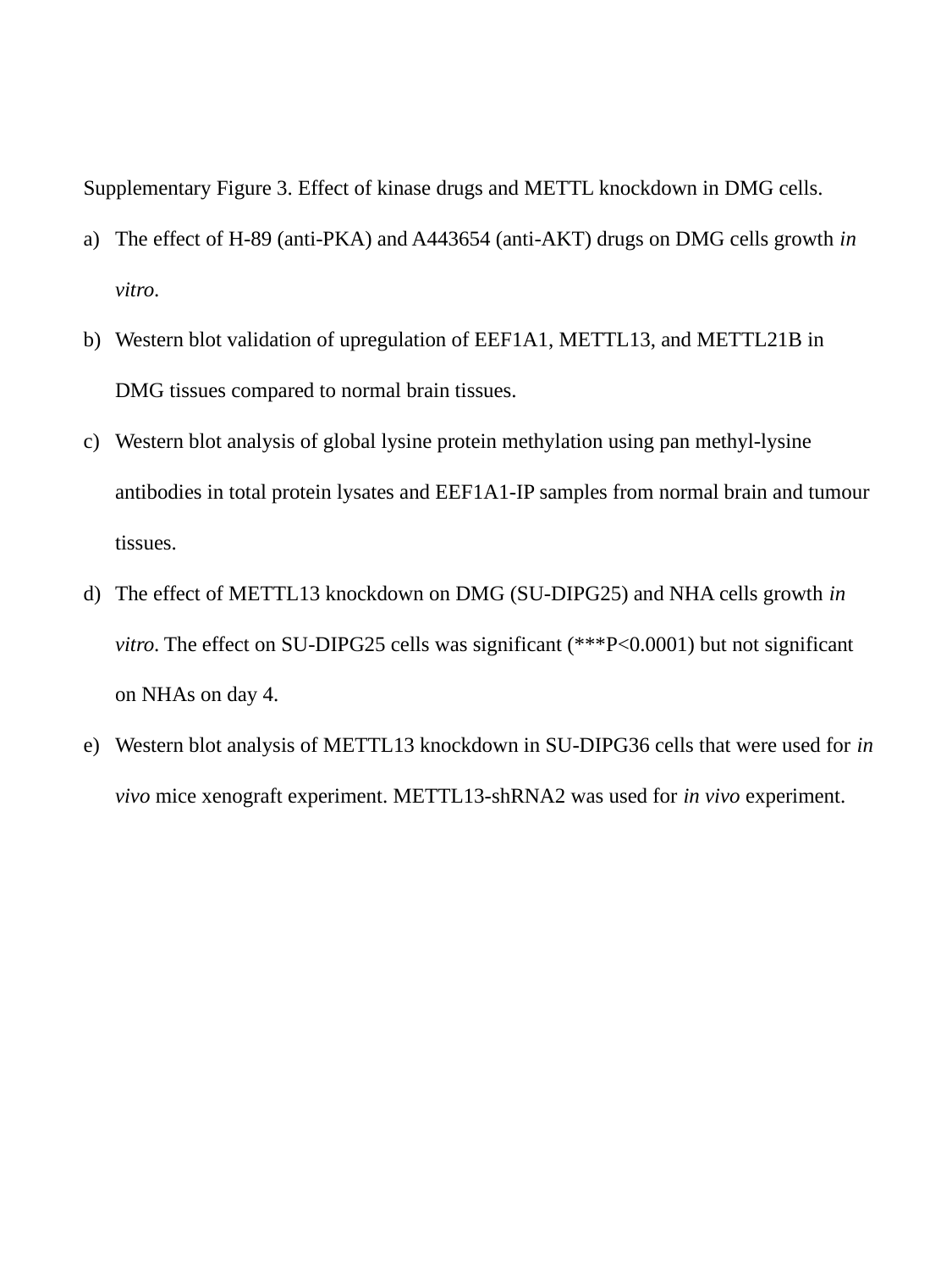

Supplementary Figure 3. Effect of kinase drugs and METTL knockdown in DMG cells.
The effect of H-89 (anti-PKA) and A443654 (anti-AKT) drugs on DMG cells growth in vitro.
Western blot validation of upregulation of EEF1A1, METTL13, and METTL21B in DMG tissues compared to normal brain tissues.
Western blot analysis of global lysine protein methylation using pan methyl-lysine antibodies in total protein lysates and EEF1A1-IP samples from normal brain and tumour tissues.
The effect of METTL13 knockdown on DMG (SU-DIPG25) and NHA cells growth in vitro. The effect on SU-DIPG25 cells was significant (***P<0.0001) but not significant on NHAs on day 4.
Western blot analysis of METTL13 knockdown in SU-DIPG36 cells that were used for in vivo mice xenograft experiment. METTL13-shRNA2 was used for in vivo experiment.

## Slide 7
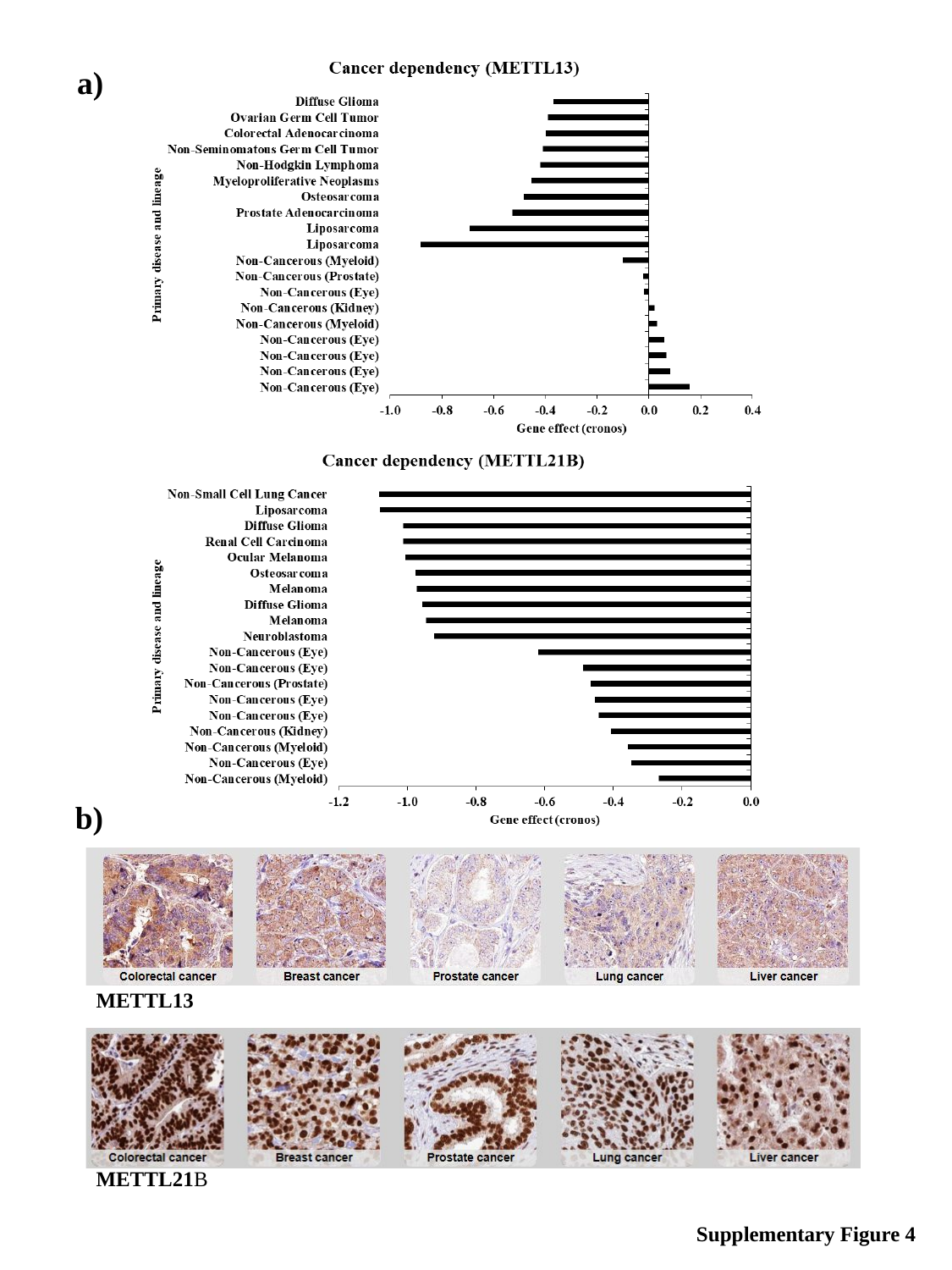

a)
b)
METTL13
METTL21B
Supplementary Figure 4

## Slide 8
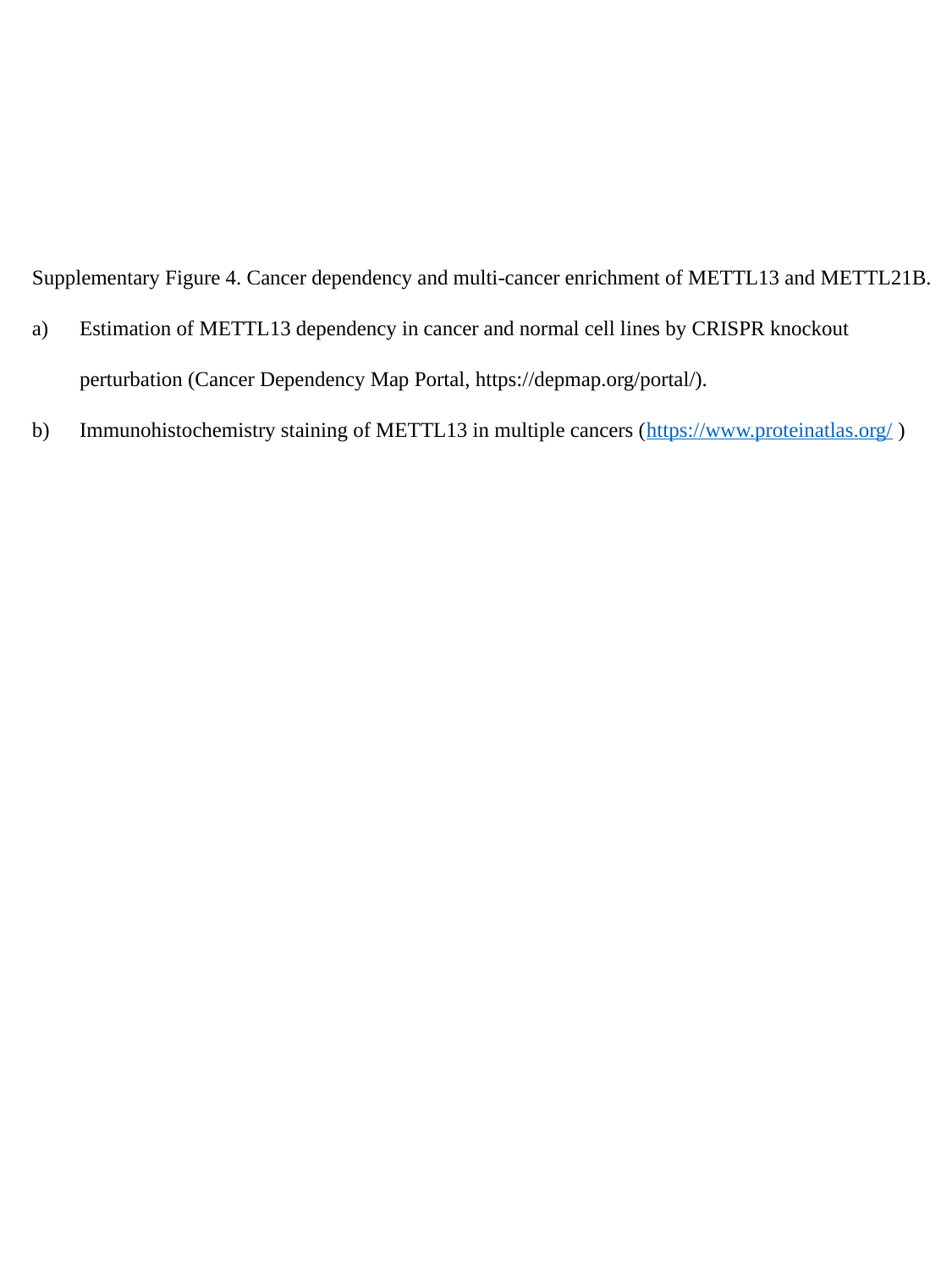

Supplementary Figure 4. Cancer dependency and multi-cancer enrichment of METTL13 and METTL21B.
Estimation of METTL13 dependency in cancer and normal cell lines by CRISPR knockout perturbation (Cancer Dependency Map Portal, https://depmap.org/portal/).
 Immunohistochemistry staining of METTL13 in multiple cancers (https://www.proteinatlas.org/ )

## Slide 9
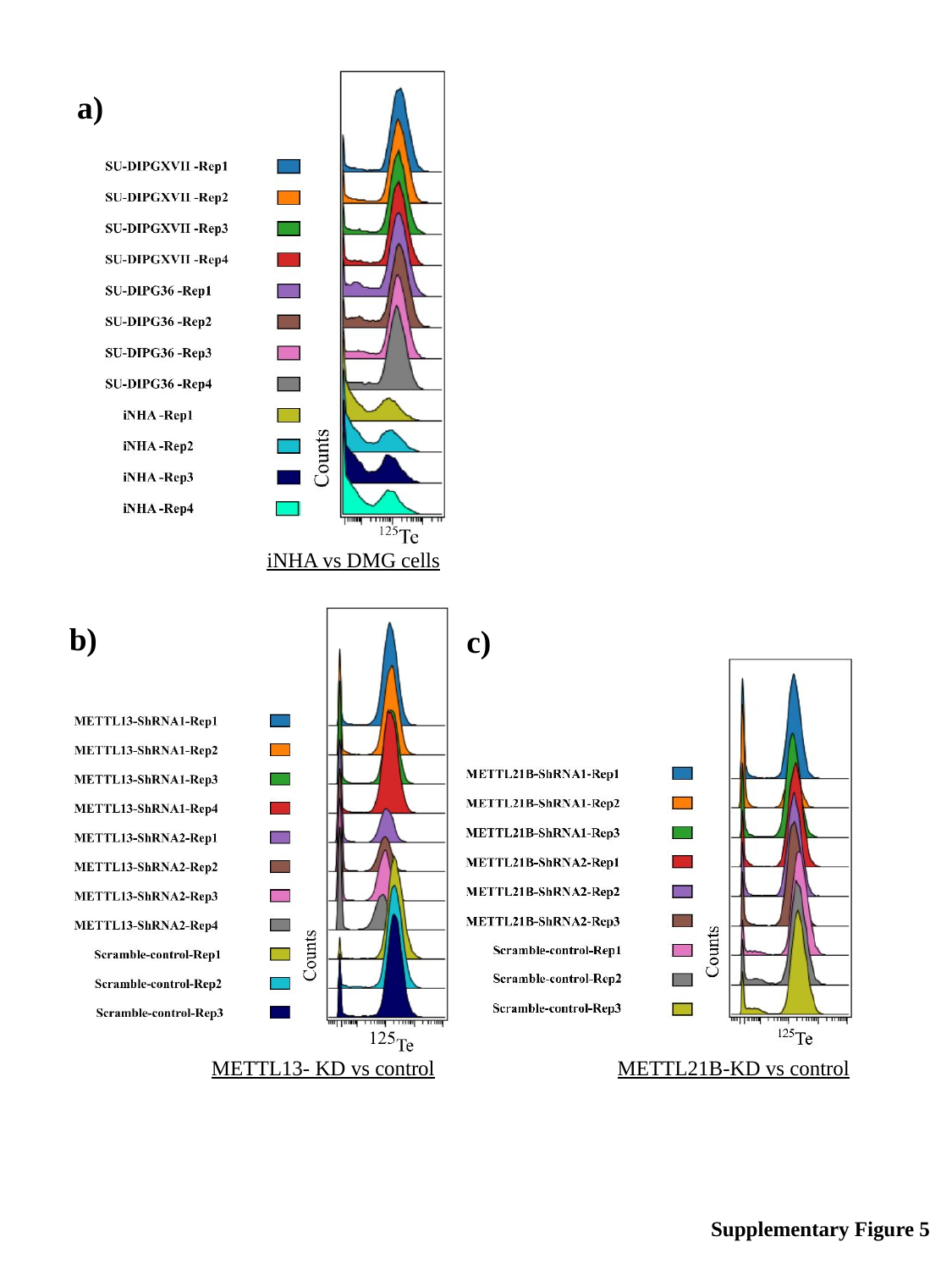

a)
iNHA vs DMG cells
b)
c)
METTL13- KD vs control
METTL21B-KD vs control
Supplementary Figure 5

## Slide 10
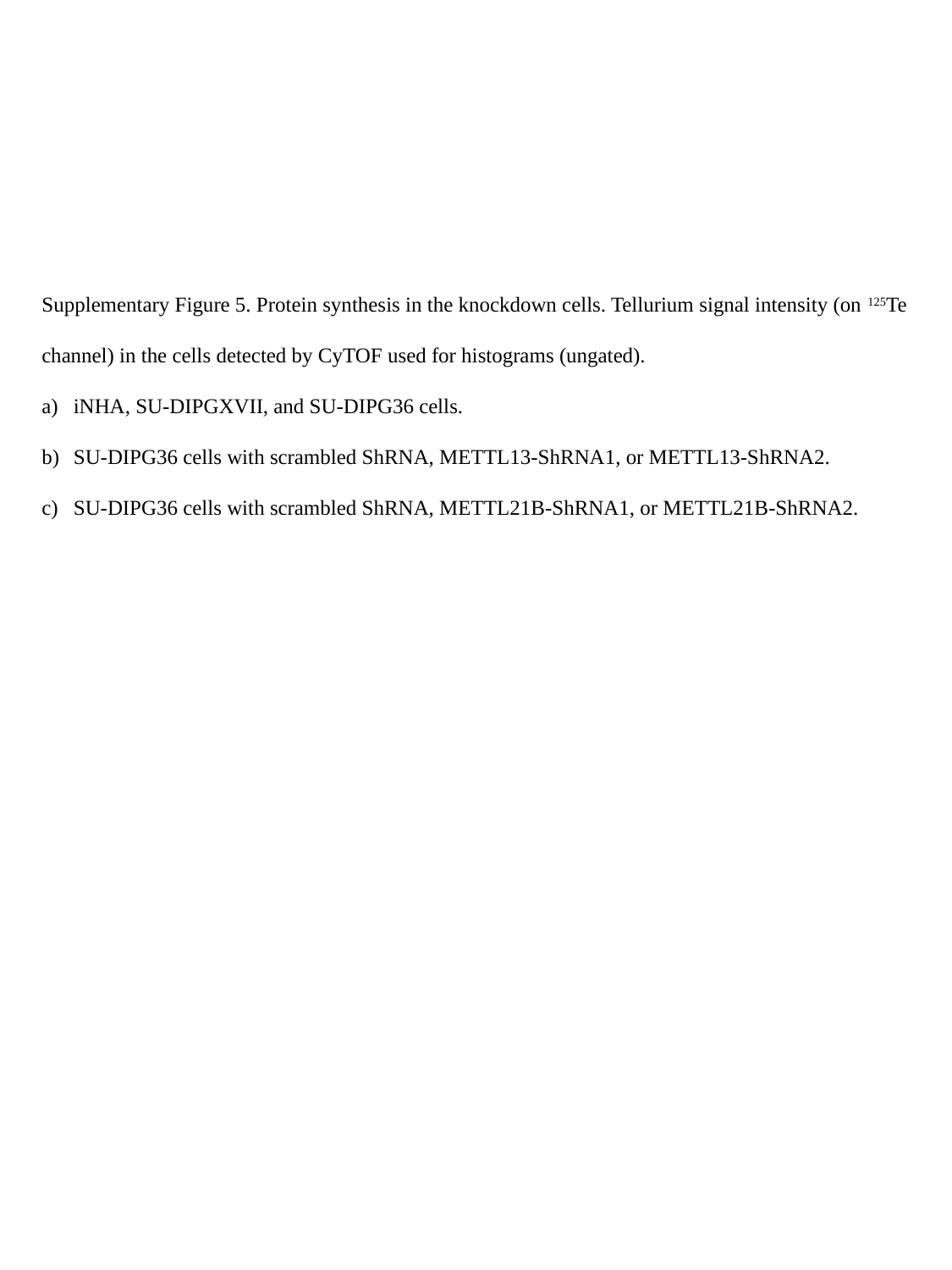

Supplementary Figure 5. Protein synthesis in the knockdown cells. Tellurium signal intensity (on 125Te channel) in the cells detected by CyTOF used for histograms (ungated).
iNHA, SU-DIPGXVII, and SU-DIPG36 cells.
SU-DIPG36 cells with scrambled ShRNA, METTL13-ShRNA1, or METTL13-ShRNA2.
SU-DIPG36 cells with scrambled ShRNA, METTL21B-ShRNA1, or METTL21B-ShRNA2.

## Slide 11
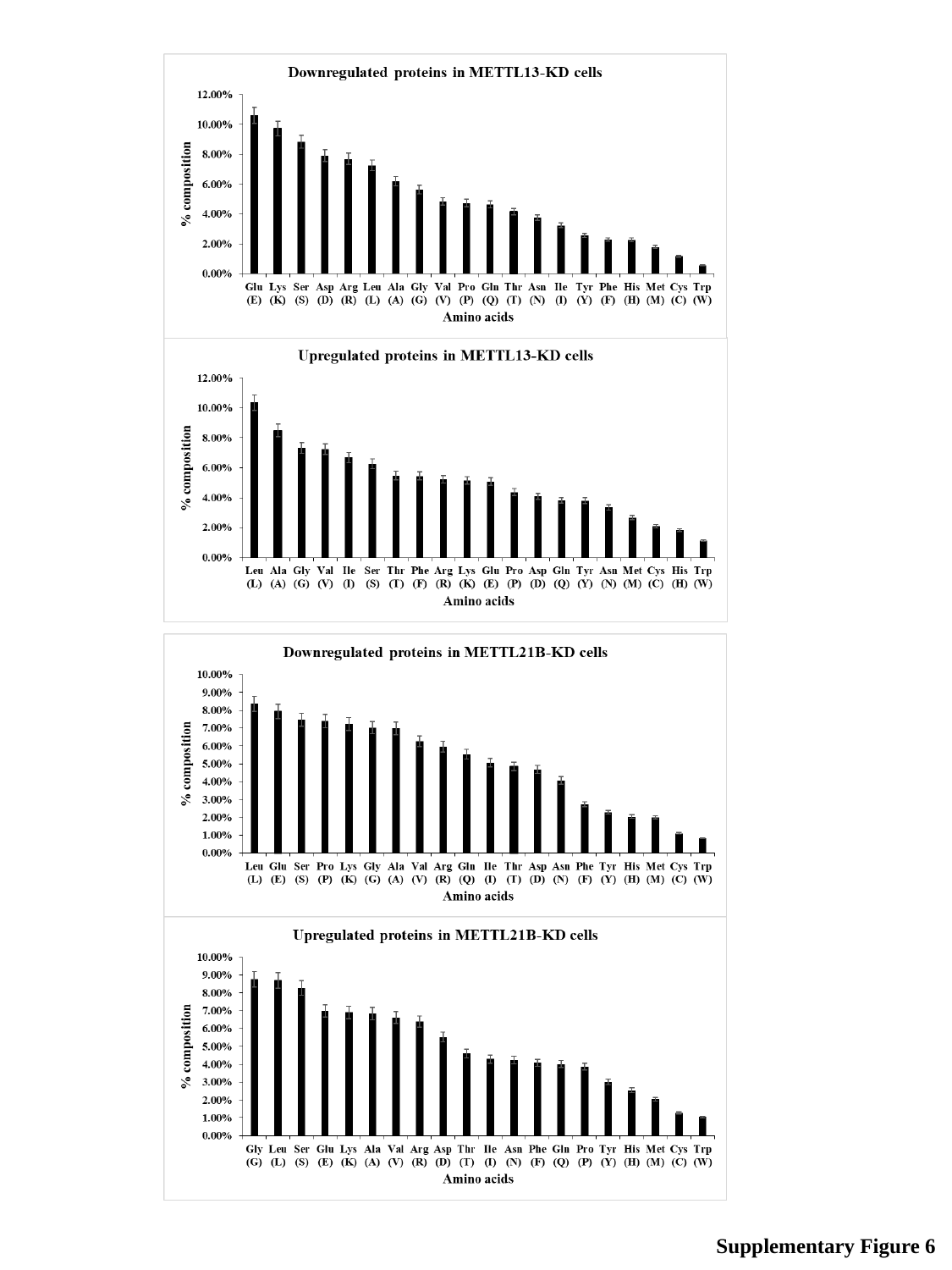

Supplementary Figure 6

## Slide 12
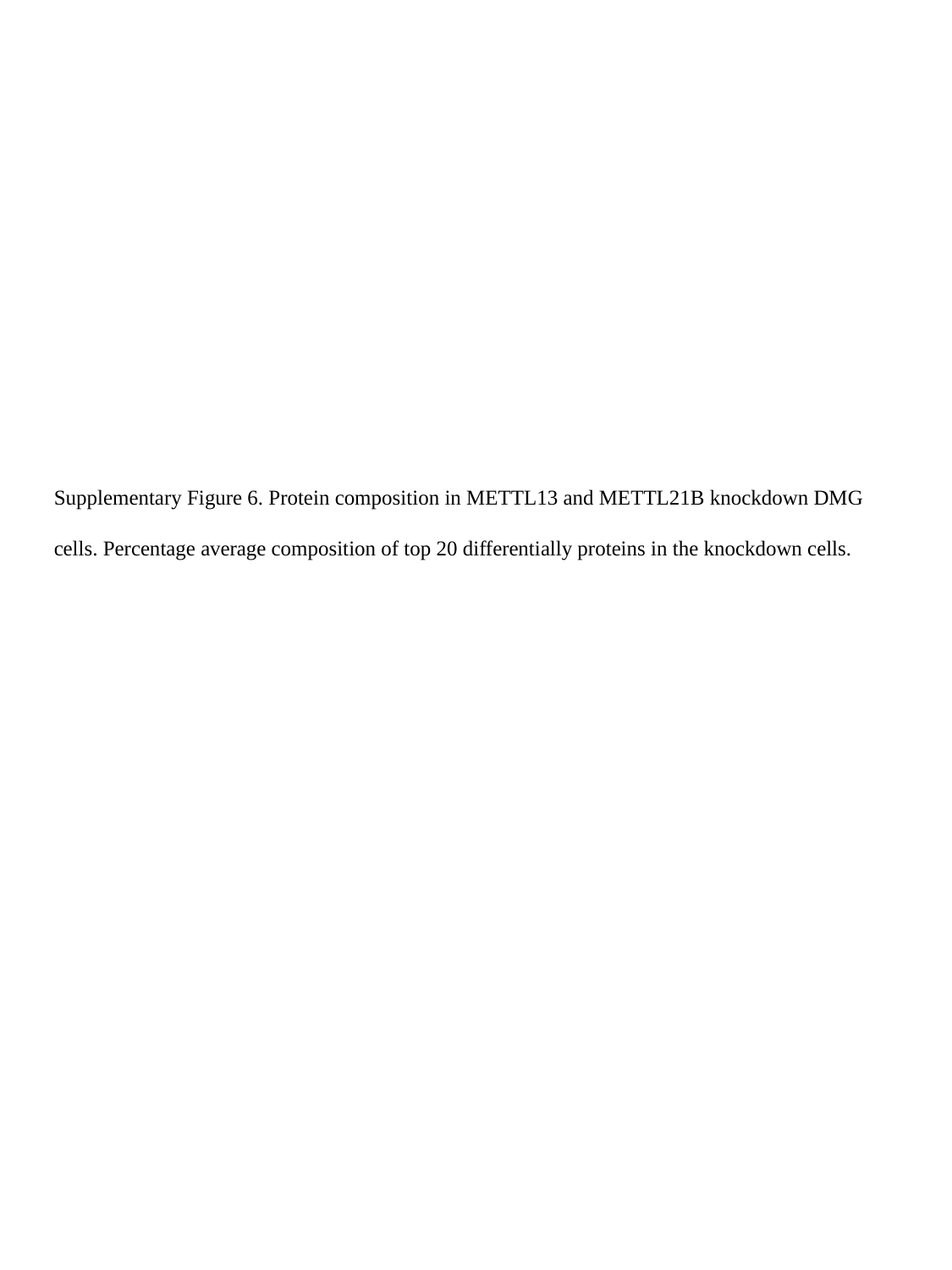

Supplementary Figure 6. Protein composition in METTL13 and METTL21B knockdown DMG cells. Percentage average composition of top 20 differentially proteins in the knockdown cells.
